# Diversification of cytokinin phosphotransfer signaling genes in *Medicago truncatula* and other legume genomes

**DOI:** 10.1186/s12864-019-5724-z

**Published:** 2019-05-14

**Authors:** Sovanna Tan, Frédéric Debellé, Pascal Gamas, Florian Frugier, Mathias Brault

**Affiliations:** 1 IPS2 (Institute of Plant Sciences Paris-Saclay), CNRS, Université Paris-Sud, Université Paris-Diderot, INRA, Université d’Evry, Université Paris-Saclay, Rue de Noetzlin, 91190 Gif-sur-Yvette, France; 2 LIPM, Université de Toulouse, INRA, CNRS, Castanet-Tolosan, France

**Keywords:** Phosphorelay, Cytokinin signaling, Histidine kinase, Response regulator, Legumes, Symbiotic nitrogen-fixing nodulation

## Abstract

**Background:**

Legumes can establish on nitrogen-deprived soils a symbiotic interaction with Rhizobia bacteria, leading to the formation of nitrogen-fixing root nodules. Cytokinin phytohormones are critical for triggering root cortical cell divisions at the onset of nodule initiation. Cytokinin signaling is based on a Two-Component System (TCS) phosphorelay cascade, involving successively Cytokinin-binding Histidine Kinase receptors, phosphorelay proteins shuttling between the cytoplasm and the nucleus, and Type-B Response Regulator (RRB) transcription factors activating the expression of cytokinin primary response genes. Among those, Type-A Response Regulators (RRA) exert a negative feedback on the TCS signaling. To determine whether the legume plant nodulation capacity is linked to specific features of TCS proteins, a genome-wide identification was performed in six legume genomes (*Cajanus cajan*, pigeonpea; *Cicer arietinum*, chickpea; *Glycine max*, soybean; *Phaseolus vulgaris*, common bean; *Lotus japonicus*; *Medicago truncatula*). The diversity of legume TCS proteins was compared to the one found in two non-nodulating species, *Arabidopsis thaliana* and *Vitis vinifera,* which are references for functional analyses of TCS components and phylogenetic analyses, respectively.

**Results:**

A striking expansion of non-canonical RRBs was identified, notably leading to the emergence of proteins where the conserved phosphor-accepting aspartate residue is replaced by a glutamate or an asparagine. *M. truncatula* genome-wide expression datasets additionally revealed that only a limited subset of cytokinin-related TCS genes is highly expressed in different organs, namely MtCHK1/MtCRE1, MtHPT1, and MtRRB3, suggesting that this “core” module potentially acts in most plant organs including nodules.

**Conclusions:**

Further functional analyses are required to determine the relevance of these numerous non-canonical TCS RRBs in symbiotic nodulation, as well as of canonical MtHPT1 and MtRRB3 core signaling elements.

**Electronic supplementary material:**

The online version of this article (10.1186/s12864-019-5724-z) contains supplementary material, which is available to authorized users.

## Background

**Fig. 1:**
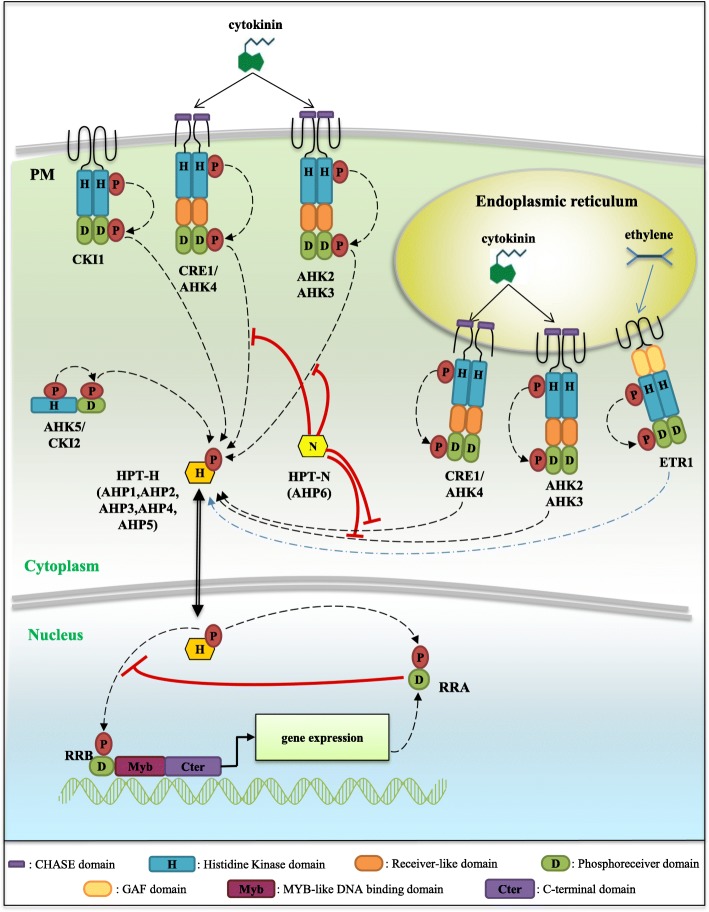
A model for the cytokinin Two Component System (TCS) phosphorelay signaling. In *Arabidopsis thaliana*, cytokinins (CKs) are perceived by histidine-kinase receptors (HK) containing two (for CRE1/AHK4) or three (for AHK2 and AHK3) transmembrane domains. CKs interact with the CHASE (Cyclases/Histidine kinases Associated Sensory Extracellular) domain of CHK receptors, inducing an autophosphorylation of a conserved histidine (H) residue. The phosphate is then transferred to a conserved aspartate (D) residue in the phosphoreceiver domain of the HKs, and to a conserved H residue of an Histidine Phosphotransfer protein (HPT-H). HPT-H proteins shuttle between the cytoplasm and the nucleus where they can transfer the phosphate group on a conserved D residue of Type-B Response Regulators (RRBs). This leads to an activation of RRBs, acting as transcription factors thanks to their Myb-like DNA binding (Myb) and transactivation domains, regulating the expression of CK-responsive genes such as Type-A Response Regulators (RRA). RRAs act as negative regulators of the CK signaling likely by competing with RRBs for phosphotransfer activation. In the HPT-N AHP6 variant, an asparagine (N) substitution of the conserved H leads to an inhibitory role on the CK signaling. In addition, CKI1, CKI2/AHK5 and the ethylene receptor ETR1 contain all domains defining an active hybrid Histidine Kinase receptor, and can interact with HPT-H, suggesting a potential function as modulators of cytokinin signaling

Cytokinin plant hormones are involved in numerous aspects of plant growth and development in relation to their environment. They regulate the balance between cell division and differentiation, and consequently plant growth, but also nutrient uptake and shoot/root metabolic relationships, as well as the adaptation toward environmental abiotic or biotic constraints [1–4]. These signals are transduced depending on a typical phosphorelay (or phosphotransfer) Two-Component System (TCS) pathway that was elucidated in the reference plant *Arabidopsis thaliana* [5, 6]. Cytokinins are perceived by a small family of Histidine Kinase receptors containing a CHASE (Cyclases/Histidine kinases Associated Sensory Extracellular) domain (CHKs, [7–9]). Cytokinin perception induces an autophosphorylation of a conserved histidine (H) residue in the kinase domain (Fig. 1). The phosphate is thereafter transferred to a conserved aspartate (D) located at the C-terminal end of the protein, in the phosphoreceiver domain. These receptors are therefore termed hybrid receptors [10]. The signal is then translocated into the nucleus, through the transfer of the phosphate group on a Histidine PhosphoTransfer protein (HPT) shuttling between the cytosol and the nucleus [11]. The phosphate is finally transmitted to type-B Response Regulators (RRBs), which are transcription factors that trigger the transcriptional activation of cytokinin primary response genes.

Features of CHK, HPT and Response Regulator (RR) proteins involved in cytokinin phosphorelay signaling have been well characterized in *A. thaliana* [3, 12]. CRE1 (Cytokinin Response 1, also named AHK4, Arabidopsis Histidine Kinase 4), was the first CHK identified following a loss-of-function genetic screen designed to search for mutants impaired in cytokinin responses [7]. Whole-genome sequencing allowed the identification of two other *A. thaliana* CHKs, AHK2 and AHK3 [7, 8]. CHKs specifically bind bioactive cytokinins thanks to their CHASE domain that is delimited by transmembrane domains [13, 14]. The three AHKs additionally contain an authentic histidine kinase domain displaying N, G1, F and G2 motifs required for the histidine kinase activity [12]. A phosphoreceiver domain is present at the C-terminal end of the proteins, containing the conserved D required for the phosphotransfer. Finally, a receiver-like domain is found between the kinase domain and the phosphoreceiver domain in all three AHKs.

Two classes of HPTs have been defined in *A. thaliana*. The first class corresponds to HPTs harboring a conserved H involved in phosphate acceptance (HPT-H, five genes in *A. thaliana*), and which are therefore able to transduce the phosphorelay initiated from CHKs towards nuclear RRBs. They are for this reason positive regulators of cytokinin signal transduction pathways [15]. In the second HPT class, the conserved H is replaced by an asparagine (N) (HPT-N, one gene in *A. thaliana*: AHP6), a residue not able to bind phosphate and therefore to mediate phosphotransfer from CHKs to RRBs. Consistently, AHP6 acts as negative regulator of cytokinin signaling notably during protoxylem formation [10].

RRs involved in cytokinin signaling are divided into two groups depending both on their structure and on their transcriptional regulation by cytokinins. All RRs have a phosphoreceiver domain structurally close to that of CHKs, with a conserved D required for the phosphotransfer. Type-A RRs (or RRAs) contain only a phosphoreceiver domain and their expression is rapidly induced by cytokinins, making these genes markers of the activation of the cytokinin primary response [16]. Genetic analyses have demonstrated that RRAs function as negative regulators of cytokinin signaling [17] (Fig. 1). Type-B RRs (or RRBs) have in addition a Myb-like DNA-binding domain, and a C-terminal transactivation domain [18]. Both RRAs and RRBs are nuclear proteins [5, 18, 19] and RRBs function as transcription factors directly controlling the expression of *RRA* genes [19–22]. In contrast to *RRAs*, *RRB* gene expression is generally not regulated by cytokinins [23]. The induction of RRAs is proposed to lead to a negative feedback competition with RRBs for accepting phosphate groups from the HPTs on the conserved D residue of their phosphoreceiver domain [17]. The RRB C-terminal transactivation domain is rich in proline (P) and glutamine (G), and its deletion impairs the ability of RRBs to promote transcriptional activation [18, 24]. In contrast, the deletion of the N-terminal phosphoreceiver domain or the replacement of the conserved D by a glutamate (E) phosphomimic residue leads to a constitutive activation of RRBs. This indicates that the phosphoreceiver domain negatively regulates RRB transcriptional activity and that this inhibitory activity can be relieved by the phosphorylation of the conserved D residue [18, 25, 26].

Other TCS elements not directly linked to cytokinin signaling exist in plants, and some of them were shown to interfere with the phosphorelay cascades activated by cytokinins. CKI1 was identified in an activation tagging genetic screen in *A. thaliana*, and its ectopic expression induced typical cytokinin responses even in the absence of exogenous cytokinins [27]. CKI1 is an authentic histidine kinase with all required features to function in a phosphorelay cascade but that does not contain a CHASE domain, and that is therefore not able to bind cytokinins [28]. When expressed in protoplasts, CKI1 could nevertheless constitutively activate cytokinin phosphorelay cascades, indicating that CKI1 may interfere with cytokinin signalling pathways [5, 29] by interacting with and phosphorylating AHPs [30, 31]. CKI1 regulates *A. thaliana* female gametogenesis and vascular tissue development [15, 29, 32], and was recently proposed to be a potential link between light and cytokinin responses to control plant development [33]. The *CKI2/AHK5* gene was identified in the same genetic screen as CKI1, and may similarly interfere with cytokinin signalling as its overexpression in *A. thaliana* calli induces cytokinin responses [27]. As CKI1, CKI2/AHK5 has authentic histidine kinase and phosphoreceiver domains but no transmembrane and CHASE domains [34]. CKI2/AHK5 is proposed to regulate abiotic and biotic responses in *A. thaliana* but no link with cytokinins has yet been established [34, 35].

Other TCS elements are involved in the perception of signals different than cytokinin, such as the *A. thaliana* AHK1 osmosensor comprising all features of an active phosphotransfer protein [36] and ethylene receptors which do not all display hallmarks of authentic histidine kinases. Indeed, several ethylene receptor proteins (ETR1, EIN4 and ETR2 in Arabidopsis; [37]) comprise from the N- to the C-terminus three transmembrane domains corresponding to the ethylene-binding domain, a GAF (cGMP-specific phosphodiesterases, Adenylyl cyclases and FhlA) domain likely involved in protein-protein interactions, a non-canonical histidine kinase domain (except *A. thaliana* ETR1 which has a canonical histidine kinase domain) and a phosphoreceiver domain. Other ethylene receptors (ERS1 and ERS2 in Arabidopsis) lack both a histidine kinase and a phosphoreceiver domain and are therefore not able to interact with HPT proteins in a phosphorelay cascade [30]. The unique Arabidopsis ethylene receptor able to function as an authentic histidine kinase receptor, ETR1, was indeed reported to physically interact with the HPT protein AHP1 and to positively regulate the ARR2 type-B RR depending on a phosphorelay cascade [30, 38–40]. However, as the *etr1* mutant can be complemented with a kinase-dead *ETR1* gene, it was concluded that the histidine kinase activity was not essential for ethylene signaling [37, 41]. A crosstalk between cytokinin and ethylene signaling may however occur through phosphorelay signaling [38, 42]. Furthermore, RRCs represent a third class of RRs beside RRAs and RRBs. RRCs contain a unique receiver domain harboring the conserved D required for phosphotransfer as in RRBs, but their sequences are phylogenetically more related to HK receiver domains than to RRA receiver domains [43]. In addition, in contrast to *RRA*s, *RRC* gene expression is not induced in response to cytokinins. Overexpression of the Arabidopsis RRC ARR22 results in a phenotype similar to the *wol* CRE1/AHK4 mutant [43]. However, it is not yet clear whether RRCs could inhibit cytokinin signaling as RRAs do. Finally, the fourth and last group of RR proteins are “clock-related RRs” containing a receiver domain where the D phospho-acceptor residue is replaced by an E, and an additional C-terminal CCT domain (for CONSTANS, CONSTANS-LIKE, and TOC1) that is involved in protein-protein interactions [44]. Such clock-RRs are involved in the control of circadian rhythms, explaining their name, and no direct interaction with the TCS cytokinin signaling has been established.

Symbiotic nodule formation results from a molecular dialog between legume roots and rhizobia. Roots release specific flavonoids, which activate the production of Nodulation factors (or Nod factors) by rhizobia. The Nod factors, once perceived in the root epidermis, trigger a genetic program leading to bacterial infection and nodule organogenesis. *Medicago truncatula* forms indeterminate-type growing nodules, with a persistent apical meristem allowing for a continuous (indeterminate) growth [45, 46]. Consequently, a metabolically active nodule comprises an apico-basal developmental gradient, consisting in an apical zone I corresponding to the meristem, followed by a plant and bacteria cell differentiation zone (zone II), and a metabolically active nitrogen-fixation zone (III) [47]. In some other legumes, such as *Lotus japonicus*, the nodule organogenesis is determinate as the meristem is not maintained, leading to the formation of round-shaped nodules. The organogenesis of both determinate and indeterminate nodules however highly relies on the activation of a cytokinin phosphorelay signaling pathway [48, 49]. Indeed, a gain of function mutation in a specific *L. japonicus* CHK most closely related to Arabidopsis AHK4/CRE1, LHK1 (Lotus Histidine Kinase 1), is necessary and sufficient to lead to spontaneous nodule formation in the absence of rhizobia [50], while loss-of-function mutants of LHK1 or MtCRE1 in *M. truncatula* are impaired in nodule formation [51–55]. Several *RRB* and *RRA* genes have been linked to nodulation based on their expression profiles [51, 56–60]. Furthermore, silencing of a subset of *RRA* genes (*MtRR4*, *MtRR5*, *MtRR9* and *MtRR11*) in *M. truncatula* roots decreases nodule formation [56].

In this study, to determine whether the nodulation capacity of legume plants may be linked to a specific subset of TCS proteins, we performed a genome-wide analysis of the *M. truncatula* genome in order to identify genes encoding putative TCS phosphorelay components associated to cytokinin signaling or potentially interfering with this pathway. We additionally proposed a unified nomenclature for *M. truncatula* accordingly to guidelines proposed in [61]. The identified TCS genes were then compared to the ones found in other legume genomes, namely *Cicer arietinum* (chickpea) forming indeterminate nodules as *M. truncatula*, and *Glycine max* (soybean), *Lotus japonicus*, *Cajanus cajan* (pigeonpea), and *Phaseolus vulgaris* (common bean) forming determinate nodules [62–66]. In addition, we included *A. thaliana* and *Vitis vinifera* as reference dicot genomes because most functional analyses of TCS genes were performed in Arabidopsis and no recent Whole Genome Duplication (WGD) occurred in *V. vinifera* [67]. Finally, extensive expression datasets available in *M. truncatula* and corresponding to different organs [68], nodule zones [69] and the early response to Nod factors in the root epidermis [60] were used to identify a subset of cytokinin signaling genes mostly linked to nodulation and therefore anticipated to act in this symbiotic interaction.

## Results

### A constrained expansion of the CHK family proteins

**Fig. 2:**
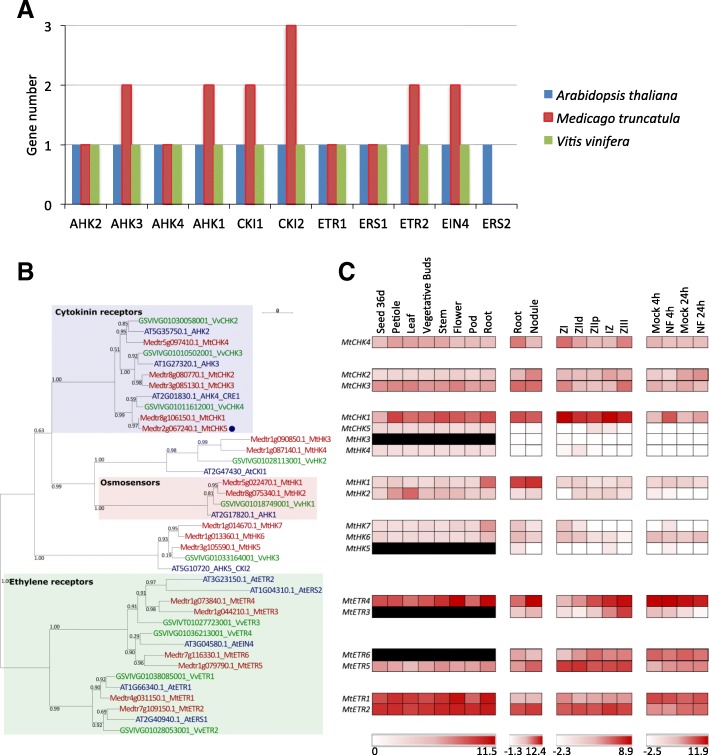
Histidine kinases in *Medicago truncatula.* **a** Number of genes encoding HKs found in the genome of *A. thaliana*, *M. truncatula* and *V. vinifera*. **b** Phylogenetic tree of HKs, based on full-length protein sequences from *A. thaliana*, *M. truncatula* and *V. vinifera*. Protein sequences were aligned with the Muscle algorithm and the phylogenic tree was built with the Seaview software package. Numbers indicate the probability for each branch. Proteins labelled with a blue dot are non-canonical HKs. **c** Heat map of the expression pattern of *M. truncatula* genes encoding HK proteins. Data were retrieved from the *M. truncatula* Gene Expression Atlas Affymetrix microarray database (MtGEA; [68]) for various organs (left panel), from [69] for roots, nodules and nodule zones (middle panels) and from [60] for root epidermis expression and response to Nod factors (NF, right panel). Meristematic zone (ZI), distal and proximal differentiation/rhizobial infection zones (ZIId and ZIIp), inter-zone (IZ) and nitrogen fixation zone (ZIII). The red/white color scale indicates log2 expression values for each heat map, which were normalized independently, with highest expression as red and lowest expression as white. A median was used as the central value and black boxes indicate that no probe was available on the microarray

**Fig. 3:**
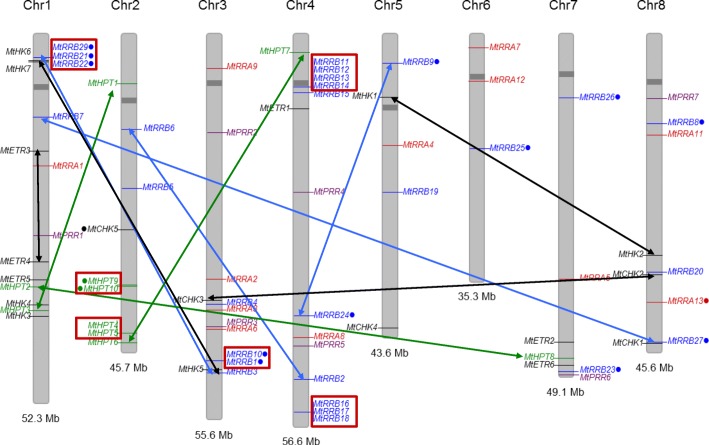
Chromosomal distribution of *M. truncatula* genes encoding TCS elements. Histidine kinase (*HK, CHK* and *ETR*) genes are indicated in black, phosphotransfer protein (*HPT*) genes in green, *RRA* genes in red, *RRB* genes in blue. Position of each gene was plotted on *M. truncatula* chromosomes using the Phenogram software. Gene names labelled with a dot encode proteins with non-canonical features. Tandem duplicated genes are shown with red boxes while genes resulting from block duplications are connected with black, green, blue and red arrows for *CHK*, *HPT*, *RRB* and *RRA* genes respectively

*M. truncatula* has one AHK4/CRE1 homolog (CHK1/CRE1), one AHK2 homolog (CHK4) and two AHK3 homologs (CHK2 and CHK3; [55]; Fig. 2a). An analysis of gene duplications indicated that the two AHK3 homologs, CHK2 and CHK3, result from block duplication (Fig. 3). An additional *M. truncatula* CHK with a truncated C-terminus region (*Medtr2g067240.1, CHK5*) was identified in this study, containing a CHASE domain delimited by two transmembrane domains associated to a partial histidine kinase domain and neither a phosphoreceiver nor a receiver-like domain (Table 1). This truncated CHK protein is most closely related to AtAHK4/CRE1 (Fig. 2 b). Despite the WGD at the origin of the *Fabaceae* family, *M. truncatula* has therefore a single additional gene encoding a full length canonical CHK compared to *V. vinifera*, as well as *A. thaliana* (Table 1). Similarly in the four other legume genomes studied, a *CHK* gene was retrieved in each AtCHK clade and only one additional *CHK* gene was detected compared to *V. vinifera* and *A. thaliana* (Additional file 1). This retained duplicated *CHK* gene is in the AHK3 clade for *C. arietinum*, as for *M. truncatula*, whereas it is in the AHK4/CRE1 clade for *C. cajan*, *L. japonicus*, and *P. vulgaris* (Additional file 2). In the soybean lineage, a more recent WGD occurred 13 Ma ago (Mya) in addition to the WGD that is common to all papilionoid legumes and which occurred about 58 Mya [70, 71]. As expected, two CHKs are retrieved in each clade (Additional files 1, 2) while an additional duplication occurred and has been retained in the AHK4 clade, indicating a cytokinin-receptor diversification as for *C. cajan*, *L. japonicus*, and *P. vulgaris*. Overall, these analyses suggest that the AHK4 duplication has been retained in a common ancestor of these four legumes and lost in *M. truncatula* and *C. arietinum* forming indeterminate nodules. Conversely, the AHK3 duplication has been conserved in a common ancestor of *M. truncatula* and *C. arietinum*. By contrast, the truncated *CHK-like* gene that is uniquely found in *M. truncatula* could be the result of a recent gene duplication, frequently observed in the *M. truncatula* genome [72].

**Table 1:** List of Two-Component-System associated proteins found in the genome of *M. truncatula*

Locus tag		Previous protein name	Protein name	Length (AA)	Arabidopsis homolog	Domains				
v4.0^a^	v5.0^b^									
*Histidine kinases*										
*Medtr5g097410*	*MtrunA17Chr5g0447641*		MtCHK4^c^	1270	AHK2	CHASE		His (H)	HATPase_c (N, G1, F, G2)	Rec (D)
*Medtr3g085130*	*MtrunA17Chr3g0122391*		MtCHK3^c^	1035	AHK3	CHASE		His (H)	HATPase_c (N, G1, F, G2)	Rec (D)
*Medtr8g080770*	*MtrunA17Chr8g0377481*		MtCHK2^c^	971	AHK3	CHASE		His (H)	HATPase_c (N, G1, F, G2)	Rec (D)
*Medtr8g106150*	*MtrunA17Chr8g0392301*	CRE1	MtCHK1^c^	1004	AHK4	CHASE		His (H)	HATPase_c (N, G1, F, G2)	Rec (D)
*Medtr2g067240*	*MtrunA17Chr2g0311191*		MtCHK5	256	AHK4-like	CHASE		His (H)	–	–
*Medtr5g022470*	*MtrunA17Chr5g0405701*		MtHK1	1201	*AHK1*			His (H)	HATPase_c (N, G1, F, G2)	Rec (D)
*Medtr8g075340*	*MtrunA17Chr8g0371601*		MtHK2	1174	*AHK1*			His (H)	HATPase_c (N, G1, F, G2)	Rec (D)
*Medtr1g090850*	*MtrunA17Chr1g0197591*		MtHK3	1051	*CKI1*			His (H)	HATPase_c (N, G1, F, G2)	Rec (D)
*Medtr1g087140*	*MtrunA17Chr1g0194741*		MtHK4	1103	*CKI1*			His (H)	HATPase_c (N, G1, F, G2)	Rec (D)
*Medtr3g105590*	*MtrunA17Chr3g0136601*		MtHK5	950	*CKI2 / AHK5*			His (H)	HATPase_c (N, G1, F, G2)	Rec (D)
*Medtr1g013360*	*MtrunA17Chr1g0150511*		MtHK6	1013	*CKI2 / AHK5*			His (H)	HATPase_c (N, G1, F, G2)	Rec (D)
*Medtr1g014670*	*MtrunA17Chr1g0150531*		MtHK7	390	*CKI2 / AHK5*			His (H)	–	–
*Medtr4g031150*	*MtrunA17Chr4g0013831*		MtETR1	791	ETR1	EBD (D,Y, I1,P, I2, C, H)	GAF	His (H)	HATPase_c (N, G1, F, G2)	Rec (D)
*Medtr7g109150*	*MtrunA17Chr7g0269631*		MtETR2	636	ERS1	EBD (D,Y, I1,P, I2, C, H)	GAF	His (H)	HATPase_c (N, G1, F, G2)	–
*Medtr1g044210*	*MtrunA17Chr1g0168161*		MtETR3	761	ETR2	EBD (D,Y, I1,P, I2, C, H)	GAF	His (−)	HATPase_c (−, −, −, −)	Rec (D)
*Medtr1g073840*	*MtrunA17Chr1g0186431*		MtETR4	760	ETR2	EBD (D,Y, I1,P, I2, C, H)	GAF	His (−)	HATPase_c (−, G1, −, −)	Rec (D)
*Medtr1g079790*	*MtrunA17Chr1g0190021*		MtETR5	763	EIN4	EBD (D,Y, I1,P, I2, C, H)	GAF	His (H)	HATPase_c (−, G1, −, G2)	Rec (D)
*Medtr7g116330*	*MtrunA17Chr7g0274831*		MtETR6	766	EIN4	EBD (D,Y, I1,P, I2, C, H)	GAF	His (H)	HATPase_c (−, G1, −, G2)	Rec (D)
*Phosphorelay-proteins*										
*Medtr1g082290*	*MtrunA17Chr1g0191351*		MtHPT2^d^	152		Hpt (H)				
*Medtr1g089130*	*MtrunA17Chr1g0196361*		MtHPT3	149		Hpt (H)				
*Medtr2g020770*	*MtrunA17Chr2g0286781*		MtHPT1^d^	148		Hpt (H)				
*Medtr2g100880*	*MtrunA17Chr2g0331011*		MtHPT4	150		Hpt (H)				
*Medtr2g100900*	*MtrunA17Chr2g0331021*		MtHPT5	150		Hpt (H)				
*Medtr7g114020*	*MtrunA17Chr7g0273321*		MtHPT8	147		Hpt (H)				
*Medtr4g010160*	*MtrunA17Chr4g0003251*		MtHPT7	169		Hpt (N)				
*Medtr2g103870*	*MtrunA17Chr2g0333131*		MtHPT6	158		Hpt (N)				
*Medtr2g085155*	*NA*		MtHPT9	151		Hpt (R)				
*Medtr2g086010*	*NA*		MtHPT10	151		Hpt (R)				
*RRAs*										
*Medtr1g049100*	*MtrunA17Chr1g0170741*		MtRRA1	198		Rec (D)				
*Medtr3g015490*	*MtrunA17Chr3g0082861*	RR9^e^	MtRRA9	164		Rec (D)				
*Medtr3g078613*	*MtrunA17Chr3g0118211*		MtRRA2	201		Rec (D)				
*Medtr3g088630*	*MtrunA17Chr3g0124861*		MtRRA3	235		Rec (D)				
*Medtr3g093860*	*MtrunA17Chr3g0128691*		MtRRA6	156		Rec (D)				
*Medtr4g106590*	*MtrunA17Chr4g0059571*	RR8^e^	MtRRA8	215		Rec (D)				
*Medtr5g036480*	*MtrunA17Chr5g0414931*	RR4^d^	MtRRA4	237		Rec (D)				
*Medtr6g007460*	*MtrunA17Chr4g0021731*		MtRRA7	184		Rec (D)				
*Medtr7g490310*	*MtrunA17Chr7g0256551*	RR5^d^	MtRRA5	239		Rec (D)				
*Medtr8g038620*	*MtrunA17Chr8g0352871*	RR11^e^	MtRRA11	177		Rec (D)				
*RRBs*										
*Medtr1g032570*	*MtrunA17Chr1g0161431*		MtRRB7	623		Rec (D)	Myb	C-ter (371 AA)		
*Medtr2g034960*	*MtrunA17Chr2g0294911*		MtRRB6	595		Rec (D)	Myb	C-ter (343 AA)		
*Medtr2g450070*	*MtrunA17Chr2g0304841*		MtRRB5	666		Rec (D)	Myb	C-ter (401 AA)		
*Medtr3g086100*	*MtrunA17Chr3g0123131*		MtRRB4	570		Rec (D)	Myb	C-ter (319 AA)		
*Medtr3g102590*	*MtrunA17Chr3g0134461*		MtRRB10	240		Rec (D)	Myb	C-ter (53 AA)		
*Medtr3g106220*	*MtrunA17Chr3g0137101*	RR3^d^	MtRRB3	645		Rec (D)	Myb	C-ter (395 AA)		
*Medtr4g021760*	*MtrunA17Chr4g0009851*		MtRRB11	312		Rec (D)	Myb	C-ter (91 AA)		
*Medtr4g021790*	*MtrunA17Chr4g0009901*		MtRRB12	274		Rec (D)	Myb	C-ter (54 AA)		
*Medtr4g021845*	*MtrunA17Chr4g0009981*		MtRRB13	311		Rec (D)	Myb	C-ter (91 AA)		
*Medtr4g021855*	*MtrunA17Chr4g0010001*		MtRRB14	300		Rec (D)	Myb	C-ter (81 AA)		
*Medtr4g023980*	*MtrunA17Chr4g0011071*		MtRRB15	201		Rec (D)	Myb	C-ter (2 AA)		
*Medtr4g121020*	*MtrunA17Chr4g0067981*	RR2^d^	MtRRB2	680		Rec (D)	Myb	C-ter (414 AA)		
*Medtr4g131570*	*MtrunA17Chr4g0074401*		MtRRB16	608		Rec (D)	Myb	C-ter (341 AA)		
*Medtr4g131580*	*MtrunA17Chr4g0074411*		MtRRB17	608		Rec (D)	Myb	C-ter (355 AA)		
*Medtr4g131600*	*MtrunA17Chr4g0074421*		MtRRB18	590		Rec (D)	Myb	C-ter (337 AA)		
*Medtr5g055260*	*MtrunA17Chr5g0423031*		MtRRB19	268		Rec (D)	Myb	C-ter (65 AA)		
*Medtr8g079940*	*MtrunA17Chr8g0376881*		MtRRB20	538		Rec (D)	Myb	C-ter (277 AA)		
*Medtr3g102600*	*MtrunA17Chr3g0134481*	RR1^d^	MtRRB1	228		Rec (E)	Myb	C-ter (56 AA)		
*Medtr4g098870*	*MtrunA17Chr4g0054981*		MtRRB24	586		Rec (E)	Myb	C-ter (193 AA)		
*Medtr5g014040*	*MtrunA17Chr5g0400241*		MtRRB9	543		Rec (E)	Myb	C-ter (180 AA)		
*Medtr6g045327*	*MtrunA17Chr6g0471041*		MtRRB25	362		Rec (E)	Myb	C-ter (102 AA)		
*Medtr7g026400*	*MtrunA17Chr7g0223971*		MtRRB26	592		Rec (E)	Myb	C-ter (393 AA)		
*Medtr8g032710*	*MtrunA17Chr8g0350761*		MtRRB8	476		Rec (E)	Myb	C-ter (203 AA)		
*Medtr8g105600*	*MtrunA17Chr8g0391911*		MtRRB27	764		Rec (E)	Myb	C-ter (519 AA)		
*Medtr0450s0040*	*MtrunA17Chr6g0473731*		MtRRB28	537		Rec (E)	Myb	C-ter (316 AA)		
*Medtr6g016850*	*MtrunA17Chr6g0456631*		MtRR31	171		Rec (E)	–	C-ter (AA)		
*Medtr8g093040*	*MtrunA17Chr8g0383961*		MtRR32	264		Rec (E)	–	C-ter (AA)		
*Medtr1g013160*	*MtrunA17Chr1g0149831*		MtRRB29	326		Rec (N)	Myb	C-ter (53 AA)		
*Medtr1g013170*	*MtrunA17Chr1g0149841*		MtRRB21	497		Rec (N)	Myb	C-ter (254 AA)		
*Medtr1g013180*	*MtrunA17Chr1g0149851*		MtRRB22	530		Rec (N)	Myb	C-ter (287 AA)		
*Medtr7g117705*	*MtrunA17Chr7g0275891*		MtRRB23	336		Rec (N)	Myb	C-ter (93 AA)		
*NA*	*NA*		MtRRB30	293		Rec (N)	Myb	C-ter (49 AA)		
*Clock-RRs*										
*Medtr1g067110*	*MtrunA17Chr1g0181811*		MtPRR1	744		Rec (E)	CCT			
*Medtr3g037390*	*MtrunA17Chr3g0091641*		MtPRR2	575		Rec (E)	CCT			
*Medtr3g092780*	*MtrunA17Chr3g0127941*		MtPRR3	685		Rec (E)	CCT			
*Medtr4g061360*	*MtrunA17Chr4g0030271*		MtPRR4	796		Rec (E)	CCT			
*Medtr4g108880*	*MtrunA17Chr4g0061021*		MtPRR5	630		Rec (E)	CCT			
*Medtr7g118260*	*MtrunA17Chr7g0276361*		MtPRR6	559		Rec (E)	CCT			
*Medtr8g024260*	*MtrunA17Chr8g0345901*		MtPRR7	585		Rec (E)	CCT			

For each gene, the current protein name, as well as a previously published name when available, the protein length, the *A. thaliana* most closely related protein, and the conserved protein domains are listed. ^a^ [73], ^b^ [74], ^c^ [55], ^d^ [51] and ^e^ [56]. NA: Not Annotated

In *M. truncatula*, *CHK1/CRE1* is the most highly expressed *CHK* gene in the different organs (Fig. 2c). All genuine *CHK* genes are expressed in roots and nodules. *CHK1/CRE1* is upregulated in response to Nod factors in the root epidermis, in contrast to other *MtCHK* genes that are expressed but not strongly regulated (Fig. 2c; [60]). Considering the *M. truncatula* AHK3 homolog pair, *CHK2* shows a weaker expression than *CHK3* in the different organs analyzed (Figs. 2 and 3). The expression of the *CHK5 CHK-like* gene is as well weak in the different organs analyzed (Fig. 2c; [60, 68, 69]). The five *CHK* genes are expressed in the different nodule zones, redundantly in the apical meristem except *CHK5*.

To determine if the *CHK* genes loss following WGD is specific of this HK subset, we also analyzed the diversification of HKs involved in ethylene perception. Compared to *V. vinifera* and *A. thaliana*, the *M. truncatula* genome contains two and one additional ethylene receptor genes, respectively. In *M. truncatula*, both *ETR2* and *EIN4* genes are duplicated whereas in *A. thaliana* only *ETR2* is duplicated, leading to the emergence of the ERS2 variant that lacks a receiver domain (Table 1; Fig. 2b). A similar distribution of HK ethylene receptors is observed in the other five legume genomes analyzed (Additional file 3). As for CHKs, soybean has twice as many ethylene receptor genes compared to other legume genomes, consistently with its recent WGD. Overall, these analyses revealed that similarly to CHKs, most of the ethylene-related *HK* genes were not retained in the different legume genomes analyzed, indicating that this feature is not specific for CK perception.

Finally, regarding HKs that may interfere with cytokinin TCS phosphorelay signaling, *AHK1*, *CKI1* and *CKI2/AHK5* genes exist in two copies in *M. truncatula* (respectively named *MtHK1–2*, *MtHK3–4* and *MtHK5–6* following [61]) and other genomes analyzed, in accordance with the legume WGD, except for CKI1 in *G. max* and *P. vulgaris* (Fig. 2b; Additional files 2, 4, 5, 6). For CKI2/AHK5, a third gene (*MtHK7*) exists specifically in *M. truncatula* but is predicted to encode a truncated protein with neither a complete histidine domain nor a phosphoreceiver domain. This third gene could result from local gene duplication since MtHK6 and MtHK7 have close locations on chromosome 1 (Fig. 3). Among these HKs, only *MtHK1* in the AtAHK1 clade, the canonical *MtHK6* gene and the non-canonical *MtHK7* gene in the CKI2/AHK5 clade, are expressed in roots and/or nodules (Fig. 2c).

### Within HPTs, only HPT1 is strongly expressed in different *M. truncatula* organs

**Fig. 4:**
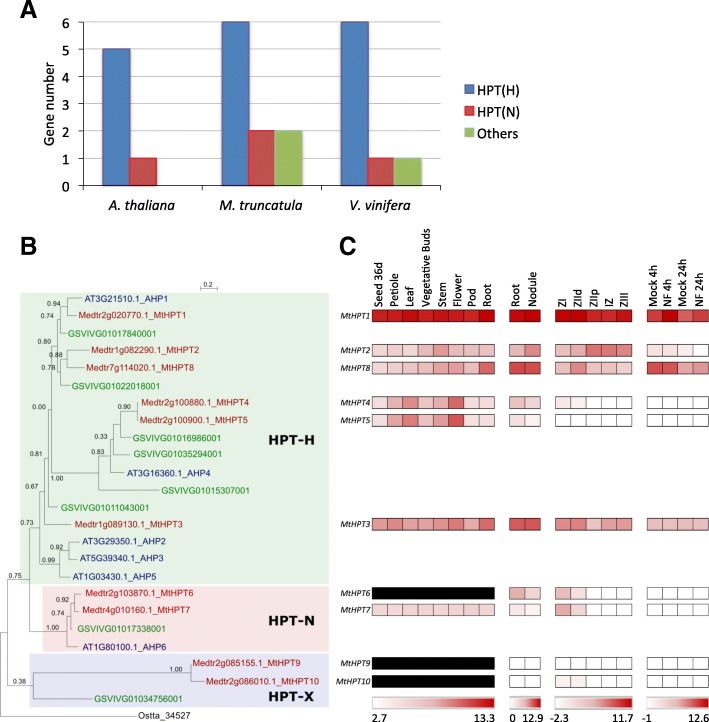
HPT proteins in *Medicago truncatula.* **a** Number of genes encoding HPT-H (blue bars), HPT-N (red bars) and non-canonical HPTs (green bars) found in the genome of *A. thaliana*, *M. truncatula* and *V. vinifera*. **b** Phylogenetic tree of HPTs, based on full-length proteins from *A. thaliana*, *M. truncatula* and *V. vinifera*. Protein sequences were aligned with the Muscle algorithm and the phylogenic tree was built with the Seaview software package. Numbers indicate the probability for each branch. The tree was rooted on the HPT Ostta_34,527 from *Ostreococcus tauri* [75]. Blue dots indicate non-canonical HPTs. **c** Heat map of the expression pattern of *M. truncatula* genes encoding HPT proteins. Data were retrieved from the *M. truncatula* Gene Expression Atlas Affymetrix microarray database (MtGEA; [68]) for various organs (left panel), from [69] for roots, nodules and nodule zones (middle panels) and from [60] for root epidermis expression and response to Nod factors (NF, right panel). Meristematic zone (ZI), distal and proximal differentiation/rhizobial infection zones (ZIId and ZIIp), inter-zone (IZ) and fixation zone (ZIII). Dashes indicate that there is no probe available on the Affymetrix *M. truncatula* chip [68]. The red/white color scale indicates log2 expression values for each heat map, which were normalized independently, with highest expression as red and lowest expression as white. A median was used as the central value and black boxes indicate that no probe was available on the microarray

*V. vinifera* and *A. thaliana* genomes contain respectively eight and six genes encoding HPT proteins while *M. truncatula* has 10 genes (Table 1; Fig. 4a). A similar number of genes (five or six) encoding HPT-H phosphoproteins is retrieved in these three genomes, whereas two HPT-N genes were identified in *M. truncatula* vs one in *A. thaliana* and *V. vinifera* (Table 1, Fig. 4a). Besides, *V. vinifera* and *M. truncatula* have additional genes encoding non-canonical HPT proteins, respectively one and two, where the conserved H is replaced by an arginine (R) for *M. truncatula* and an isoleucine (I) for *V. vinifera* (Table 1, Additional file 7). These non-canonical HPT proteins are grouped in a specific clade of the HPT protein phylogenetic tree (collectively named HPT-X; Fig. 4b) and also clustered on the *M. truncatula* chromosome 2 (Fig. 3). In the other legume genomes, a similar number of HPT-H and HPT-N genes were identified as in *M. truncatula* (Additional file 7). Additional non-canonical HPTs were also retrieved: HPT-R variants in *C. cajan*, *G. max* and *P. vulgaris*; and two HPT-L variants in *G. max* and one in *L. japonicus*, respectively (Additional files 7, 8). At the predicted phosphoacceptor position (H77 in MtHPT3) in the 78 HPTs identified in this study, H (65%) or N (19%) residues are found in 84% of HTPs (Additional file 9A). Regarding H positions different than the predicted phosphoacceptor site, only 2 to 53% contains a H or a N residue within the 78 HPT proteins analyzed. This suggests that the rate of substitution of the H involved in phosphotransfer is reduced compared to other H residues.

Among the 10 *M. truncatula HPT* genes, *MtHPT1* has the highest expression in all plant organs studied, including nodules where the expression is maximal in the meristematic zone I and the distal part of differentiation/rhizobial infection zone II (Fig. 4c). *MtHPT1* expression is also induced by NFs in the root epidermis. *MtHPT3, 4* and *5* are expressed in leaves and flowers, *MtHPT3* and *8* in roots and nodules, even though their expression is not regulated by NFs in the root epidermis (Fig. 4c). Genes encoding non-canonical HPT-N (*MtHPT6* and *MtHPT7*) and HPT-R (*MtHPT9* and *MtHPT10*) are weakly expressed whatever the organ considered (Fig. 4c) potentially because of an expression pattern limited to a small number of cells.

### Expansion of non-canonical RRBs in legume genomes

**Fig. 5:**
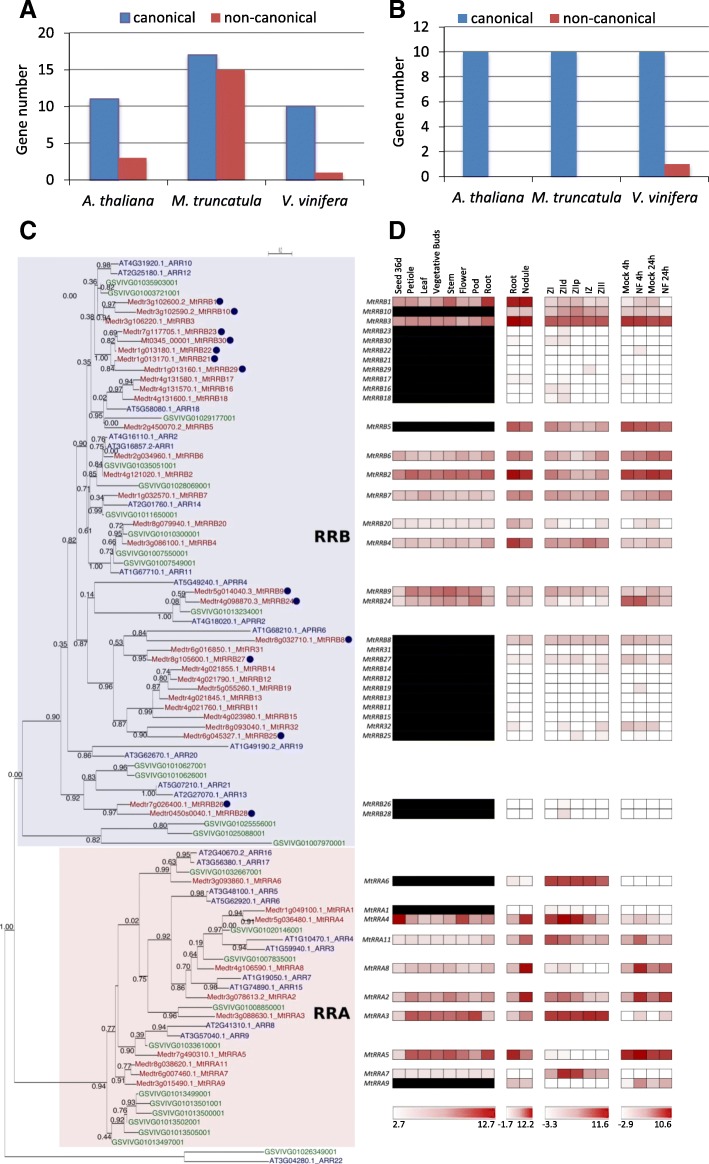
Type-A and Type-B Response Regulators in *Medicago truncatula.* **a**-**b** Number of genes encoding RRBs (**a**) and RRAs (**b**), respectively, identified in the genome of *A. thaliana*, *M. truncatula* and *V. vinifera*. **c** Phylogenetic tree of RRs, based on full-length proteins from *A. thaliana*, *M. truncatula* and *V. vinifera*. Protein sequences were aligned with the Muscle algorithm and the phylogenic tree was built with the Seaview software package. Numbers indicate the probability for each branch. The tree was rooted on the ARR22 from *A. thaliana* [75]. Proteins labelled with a blue dot are non-canonical RRs. **d** Heat map of the expression pattern of *M. truncatula* genes encoding RR proteins. Data were retrieved from the *M. truncatula* Gene Expression Atlas Affymetrix microarray database (MtGEA; [68]) for various organs (right panels), from [69] for roots, nodules and nodule zones (middle panels), and from [60] for root epidermis expression and response to Nod factors (NF, right panels). Meristematic zone (ZI), distal and proximal differentiation/rhizobial infection zones (ZIId and ZIIp), inter-zone (IZ) and fixation zone (ZIII). Dashes indicate that there is no probe available on the Affymetrix *M. truncatula* chip [68]. The red/white color scale indicates log2 expression values for each heat map, which were normalized independently, with highest expression as red and lowest expression as white. A median was used as the central value and black boxes indicate that no probe was available on the microarray

The *M. truncatula* genome contains 32 predicted proteins grouping with *A. thaliana* and *V. vinifera* RRBs (Table 1, Fig. 5a, c), i.e. about three times more than *V. vinifera* and two times more than *A. thaliana*. Seventeen of them encode authentic RRBs (i.e. with a phosphoreceiver domain containing a conserved phosphoacceptor D residue, a DNA-binding domain, and a transactivation domain) vs 10 in *V. vinifera* and 11 in *A. thaliana*. The remaining 15 *M. truncatula* RRBs are non-canonical, the conserved D being replaced by E or N in most cases (Table 1). Seven MtRRBs seem to have a transactivation domain shorter than 100 residues, vs 200–500 residues in authentic RRBs (Table 1). *V. vinifera* and *A. thaliana* genomes encode respectively only one and three non-canonical *RRB* genes (with the D replaced by either an E, N or Q residue; Additional file 10), indicating that there has been comparatively a strong expansion of non-canonical RRBs in *M. truncatula*. This expansion likely results from tandem duplications since these proteins are clustered in the phylogenetic tree in clades where *V. vinifera* or *A. thaliana* RRB proteins are absent (Fig. 5c), and most of them are also physically clustered in four blocks on *M. truncatula* chromosomes 1, 3 and 4 (Fig. 3). Block duplications are in addition observed, corresponding to four pairs of genes: *MtRRB3/MtRRB29*, *MtRRB7/MtRRB27*, *MtRRB2/MtRRB6*, *MtRRB1/MtRRB10* (Fig. 3). In two of these block-duplicated pairs, one of the paralogs has lost the conserved D residue required for phosphotransfer (Figs. 3 and 5; Table 1). Two *M. truncatula* RRs (MtRR31 and MtRR32) grouping with authentic RRBs consist of a single receiver domain with neither a DNA binding domain nor a trans-activation domain. These two proteins therefore resemble RRA proteins (see below) but in contrast to authentic RRA they have an E instead of the conserved D residue associated to the phosphotransfer. The other legume genomes analyzed have roughly a similar number of authentic RRBs as *V. vinifera* and *A. thaliana*, but the number of non-canonical *RRB* genes is also increased, while this number remains similar also in *G. max* despite its additional WGD (Additional files 10, 11). Interestingly, in all legume genomes analyzed, non-canonical RRBs have conserved D to E or N substitutions. We analyzed in the 138 RRBs identified in this study the substitution rates for different D positions within or outside the predicted phosphoacceptor site (D64 in MtRRB3) (Additional file 9B). In 95% of the phosphoacceptor sites, the position was occupied by a D (64%), an E (25%) or an N (6%), (Additional file 9B), while at D positions outside of this site (eg D192 in MtRRB3) 30% of the 138 RRBs had a residue different than D, E, or N. This suggests that, as for HPT proteins, the predicted phosphoacceptor site has a reduced substitution rate.

**Fig. 6:**
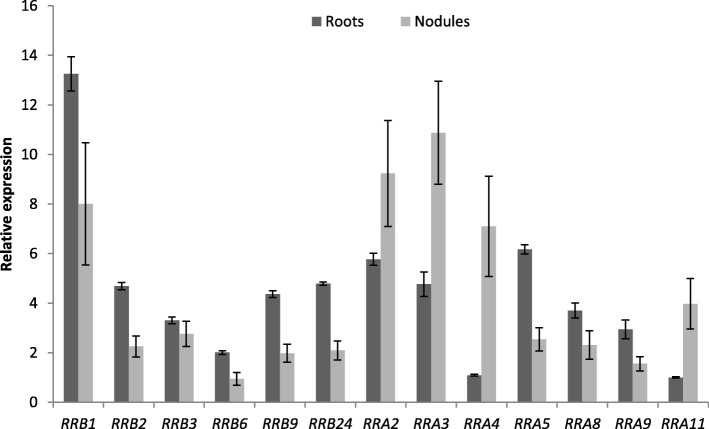
Expression of selected *RRB* and *RRA* cytokinin signaling related genes in roots and nodules. Real-time RT-PCR analysis of RRB and RRA encoding gene expression in non-inoculated roots or in nodules (8 days after inoculation with *S. meliloti*), selected based on their detectable expression and/or their differential expression level in roots versus nodules in transcriptomic datasets. The value of the lowliest expressed gene in non-inoculated roots (*MtRRA11*) was arbitrarily set to 1 as a calibrator. Data were normalized using the mean expression of two reference genes, and error bars represent Standard Deviations from one representative biological replicate out of two. RRB, Type-B Response Regulator; RRA, Type-A Response Regulator

Expression of 27 of the 32 *M. truncatula* *RRB* genes was detected in the transcriptomic datasets analyzed, including 14 (out of 18) canonical and 13 (out of 14) non-canonical RRBs, in different plant organs including nodules (Fig. 5d). Three *RRB* genes, one non-canonical D-to-E RRB (*MtRRB1*) and two canonical (*MtRRB2* and *MtRRB3*), show the highest expression level in roots and nodules. The expression of other non-canonical *RRB* genes can be detected in roots and nodules, corresponding to three D-to-E RRBs and the one truncated RRB lacking the Myb domain (Fig. 5d). Beside *MtRRB2* and *MtRRB3*, most other authentic RRBs are expressed in different organs and notably in roots and in the different nodule zones. The expression level in roots and nodules of *MtRRB* genes independently tested by real-time RT-PCR revealed similar results as transcriptomic datasets (Fig. 6). Considering the origin of these genes, tandem duplicated genes are weakly expressed with the exception of MtRRB1 and MtRRB10 (Figs. 3 and 5d), whereas for block-duplicated genes, in each of the three pairs identified, one of the paralogs shows a weaker expression than the other duplicated gene (Figs. 3 and 5d).

### A constrained expansion and structure conservation of the RRA family

The *M. truncatula* genome contains 10 genes encoding RRA proteins, similarly to *A. thaliana* and *V. vinifera* genomes that contain respectively 10 and 11 *RRA* genes (Table 1; Fig. 5b). Among the six legume genomes studied here, six genes encoding potential RRC proteins were found in *G. max* (Additional file 12). The *M. truncatula* genome contains seven genes encoding clock-RRs, i.e. two more genes that *V. vinifera* and *A. thaliana* (Additional file 13). All *RRA* and *clock-RR* genes are expressed in most organs and in all nodule zones (Fig. 5d).

All RRA proteins have a canonical structure and display the conserved D required to act in a phosphorelay cascade. Among all these genes, only *MtRRA2* and *MtRRA8* result from block duplication (Figs. 3 and 5d). Other legume genomes analyzed also contains between 8 and 14 *RRA* genes while *G. max* has 20 genes due to its specific WGD, all being authentic RRAs (Additional files 11, 14). Thus, in contrast to RRBs, the ancestral legume WGD was not followed by an expansion of *RRA* genes, and the additional soybean WGD was not followed by a global loss of RRAs.

Expression of *RRA* genes is detected in all organs analyzed. *MtRRA2/3/4/8*/*11* transcripts are more abundant in nodules than in roots whereas *MtRRA5* shows an opposite expression pattern (Fig. 5d). In nodules, the expression of different *RRA* genes is detected in the different zones, *MtRRA4* being the most expressed, mainly in the differentiation/rhizobial infection zone II. The expression level in roots and nodules of *MtRRA* genes independently tested by real-time RT-PCR overall revealed similar results as transcriptomic datasets (Fig. 6). A subset of *RRA* genes (*MtRRA2/5/8/9/11*) is expressed in the root epidermis and induced by Nod factors consistently with cytokinin signaling pathways being active in the epidermis (Fig. 5d; [56, 59, 60]). In contrast, *MtRRA4* is not regulated by NFs in the root epidermis, suggesting that it may be more related to nodule organogenesis in the root cortex as previously proposed (Fig. 5d; [53]). We finally searched in the promoter of all these *M. truncatula RRA* genes the number of “AGATHY” cytokinin responsive *cis-*elements (Additional file 15) proposed in *A. thaliana* to be directly regulated by RRB transcription factors, and therefore cytokinin signaling [76]. Between 6 to 21 AGATHY motifs per 2.5 kb of promoter regions were identified; this number was however neither strictly correlated to the strength of gene expression in roots or during nodulation, nor in relation to root and nodule expression clusters identified using a hierarchical clustering approach (Additional file 16).

## Discussion

### A function for non-canonical TCS variants

The most striking characteristic of the legume cytokinin signaling gene families is an expansion of TCS proteins with non-canonical features, as compared to *V. vinifera* and *A. thaliana.* This is especially obvious for MtRRBs for which almost half of the genes encode non-canonical transcription factors. About one third of these non-canonical RRBs show a detectable expression in the conditions analyzed. The expression of the remaining genes may take place in other conditions or be restricted to a few cells, making its detection difficult, or alternatively may be disappearing because of pseudogenization [77]. A recent study of TCS in various plants but not legumes revealed that among TCS families expansion mostly occurs in the RR gene family, in agreement with our results [78]. An expansion of non-canonical RRBs was however not reported, even in more detailed studies focused on rice and poplar genomes [79, 80]. Further dedicated studies would be needed to definitively establish whether this variant enrichment is legume-specific or not.

Considering all non-canonical RRB and HPT proteins identified within the six selected legume genomes, a striking observation is that the conserved residue required for the phosphotransfer (a D for RRBs, or an H for HPTs) is mostly replaced by a residue a priori unable to participate in the phosphotransfer but restricted to a few amino acids. This substitution might relate to a functional diversification of these proteins: indeed, the conserved D-to-E substitution frequently observed in legumes at the RRB predicted phosphoreceiver site has been shown to maintain RRB transcription factors in a constitutive active state [5, 26, 81]. In contrast, the H-to-N substitution identified initially in the Arabidopsis AHP6 protein at the phosphoacceptor site impedes its activation by phosphotransfer [10]. Specific functional variants may have then arisen in legume genomes following WGD, block, and/or tandem gene duplication events. In addition to the loss of conserved residues required for phosphotransfer regulation, D for RRBs or H for HPT proteins, most of these atypical duplicated genes display a very weak or narrow expression pattern. This is especially noticeable for block-duplicated genes where one of the two paralog shows a strong expression pattern while the second can be almost not expressed. Indeed, beside cases where one gene is retained while the second duplicated gene is lost or pseudogenized, an alternative fate is that both genes remain functional, either with a shared function or with a neofunctionalization [70]. In *A. thaliana* for example, single, double and triple mutants affecting the canonical RRBs proteins ARR1, ARR10 and ARR12 showed a progressive increase in the number of deregulated target genes, indicating that gene duplication increases both the diversity of target genes and the robustness of their regulation [82]. In the case of the AHP6 non-canonical HPT (HPT-N), the phosphotransfer capacity is lost leading to an opposite function than canonical HPTs as a negative regulator of cytokinin phosphotransfer signaling [10]. Specific expansion of a subset of expressed non-canonical D-to-N or D-to-E RRBs in different legume genomes suggests that some of these RRBs may have acquired new functions, either as inhibitors of the phosphotransfer, or as phosphotransfer-independent transcription factors that may or may not be linked to cytokinin signaling. Interestingly in Arabidopsis, the APRR2 protein is similar to authentic RRBs due to its Myb-like DNA-binding and phosphoreceiver domains, but cannot be regulated by a phosphorelay cascade since the conserved D is replaced by a E. By interacting with the calmodulin protein CML9, APRR2 seems to be involved in responses to abiotic stress and ABA signaling more than in a cytokinin-signaling pathway [83]. In tomato, an APRR2 ortholog was proposed to participate in the control fruit ripening [84], another physiological process for which a cytokinin regulation is usually not reported as critical.

The roles of such non-canonical RRs are not yet elucidated in legumes. MtRRB1 is a non-canonical RRB highly expressed in *M. truncatula* roots and nodules ([51]; this study). In contrast to authentic RRBs, MtRRB1 is predicted to be constitutively activated because of the D-to-E substitution in the predicted phosphoreceiver site. MtRRB1 can bind promoters of early nodulation genes such as *NSP2*, as well as of cytokinin primary target genes such as *RRA4*, but no nodulation phenotype was reported upon silencing by RNAi or overexpression [21]. *MtRRB1* overexpression in *A. thaliana* roots however increased root length [21], a phenotype opposite to the one expected for an authentic RRB acting as a positive regulator of cytokinin signaling, and which might suggest a negative role in this signaling pathway. As RRBs that have lost the predicted phosphoacceptor D residue are expected to be unable to be regulated by phosphotransfer, these non-canonical proteins may be activated by an alternative mode of regulation, as reported for APRR2 in Arabidopsis [83], e.g. by a binding to calmodulin, S / T phosphorylation, ubiquitination or other post-translational regulatory modifications.

### Cytokinin signaling and symbiotic nodulation: a main core signaling recruited from existing pathways?

One objective of analyzing proteins related to cytokinin signaling in legumes was to define which subsets of proteins could be linked specifically to the nitrogen-fixing symbiotic capacity of these plants, and to determine whether differences correlating with the ability to form determinate or indeterminate nodules could be identified in TCS gene families. No specific feature was highlighted concerning the ability of legumes to form indeterminate- or determinate-type, except the structuration of the CHK family. This perceived correlation may be however linked to the close relationships between the genomes analyzed, and additional phylogenetic analyses based on more diverse high-quality legume genomes, when available, would be needed to more convincingly address this issue. In addition, it remains to be tested whether differences may exist in upstream events linked to cytokinin metabolism, and/or to downstream RRB target gene regulation. The independent expression datasets analyzed in this study revealed that in each TCS protein family, a few members are more strongly expressed in nodules than others, leading to define a core symbiotic nodule cytokinin signaling module, notably highlighted by a hierarchical clustering focused on transcriptomic datasets from roots and nodules, consisting of the MtCRE1/MtCHK1 receptor, the MtHPT1 phosphotransfer protein, and the MtRRB3 transcription factor, while more variation in expression levels was observed for RRAs. The functional relevance of this core pathway remains to be evaluated, even though it is already established in different legumes that, at early symbiotic stages, the most expressed cytokinin receptor (MtCHK1/CRE1, LjLHK1, AhHK1 in *Arachis hypogea*, or AeHK1 in *Aeschynomene evenia*) gene is also the most functionally relevant for nodulation [51–55, 85, 86]. Noteworthy, the MtCHK1(MtCRE1)/MtHPT1/MtRRB3 cytokinin signaling core is also the most highly expressed in the different *M. truncatula* organs analyzed, indicating that this is not a nodule-specific cytokinin signaling module. Considering the different nodule zones defined in *M. truncatula* indeterminate nodules, no clear-cut sub-specialization of cytokinin signaling protein family members could be identified for CHKs, HPTs and RRBs, with notably MtCRE1/MtCHK1, MtHPT1 and MtRRB3 being expressed in all different zones. Therefore, the proposed “core cytokinin signaling module” may regulate processes as diverse as the maintenance of the nodule apical meristem, cell differentiation and infection by symbiotic rhizobia bacteria, and nitrogen fixation, as suggested for MtCRE1 [55]. Finally, the expression pattern of *RRA* genes shows more variation within the different nodule zones, with *MtRRA3*, *MtRRA4*, *MtRRA6* and *MtRRA7* mostly expressed in the nodule apex (zones I and II), while *MtRRA3* and *MtRRA6* are in addition expressed in the nitrogen-fixing zone (III). Strikingly, the hierarchical clustering did not reveal any cluster associating *CHK/HPT/RRB* genes with *RRA* genes, which all grouped in separated clusters. This diversity of RRA expression patterns may reflect that various mechanisms modulate cytokinin signaling depending on organs and even nodule zones, likely depending on other regulatory signals.

Finally, regarding HKs that can potentially modulate TCS cytokinin signaling in Arabidopsis [42], expression data reveal that all ethylene receptors (MtETR1–6), but also the osmosensor MtHK1 and the two CKI2 homologs MtHK6–7 have at least partially overlapping expression patterns with *CHK* and *HPT* genes in the different organs analyzed, including the different nodule zones. This suggests that these histidine kinases receptors could indeed interfere with cytokinin signaling phosphorelay as already proposed in Arabidopsis. Cytokinin and ethylene hormones are indeed both known to participate in the control of nodule initiation [49, 87]. Each of these two hormones can influence positively the accumulation and/or the response of the other [57, 88]. At the molecular level however, the ethylene-cytokinin crosstalk remains poorly described in symbiotic nodulation, and among other mechanisms, one can speculate that an interaction between the two hormones may exist at the TCS phosphorelay cascade level.

## Conclusions

In this study, we have identified all genes encoding proteins predicted to participate in or interfere with cytokinin phosphorelay signaling, and proposed for the *M. truncatula* genome a unified nomenclature accordingly to guidelines proposed in [9]. A MtCHK1(MtCRE1)/MtHPT1/MtRRB3 typical cytokinin signaling core has been defined, which is the most highly expressed in the different *M. truncatula* organs analyzed including symbiotic nodules. Whereas following the ancestral WGD associated to the papilionoid subfamily of legumes, *M. truncatula* and all other legumes analyzed have maintained a number of *CHK*, *HPT* and *RRA* genes similar as in *V. vinifera* and *A. thaliana* reference genomes, indicating a high selection after WGDs, the *RRB* gene family was systematically expanded. More strikingly, this involved an increase of TCS proteins with non-canonical features, with almost half of MtRRBs encoding non-canonical transcription factors from which one third show a detectable expression in the conditions analyzed. Further work is needed to evaluate the functionality of these variants as well as their occurrence in non-legume genomes.

## Methods

### Material, plant growth conditions and treatments

The *Medicago truncatula* Jemalong A17 genotype was used in this study. Seeds were scarified by immersion in pure sulfuric acid for 3 min, rinsed six times with water, and sterilized for 20 min in Chlorofix (8.25 mg/L. Bayrol, France). After three washes with sterilized water, seeds were sown on 1% agar plates, and stratified for 3 days at 4 °C in the dark. Germination was triggered by an overnight incubation at 24 °C in the dark. Germinated seeds were grown in vitro on a Fahraeus medium without nitrogen [89] with 1.5% bacto-agar (Gibco) in a growth chamber (16 h light at 150 μE intensity, 24 °C, 60% relative air humidity), and the *Sinorhizobium meliloti* Sm1021 strain was used to nodulate plants. Bacteria were grown overnight at 30 °C on a Yeast Extract Broth (YEB) medium. Roots were inoculated for 1 h with a bacterial suspension (OD_600nm_ = 0.05), collected and immediately frozen in liquid nitrogen for RNA extraction.

### Sequence identification, analysis and classification

To identify all TCS proteins in the different genomes selected, BlastP searches (e-value cut-off of 1.0) were performed using as queries, as suggested by [61], the receiver domain of ARR6 (At5g62920.1) for the identification of RR proteins, the histidine kinase domain of AHK4/CRE1 (At2g01830.2) for the identification of HK proteins and the HPT domain of AHP1 (At3g21510.1) for the identification of HPT proteins against the proteomes of various papilionoid legume genomes available in the Legume Information System database (LIS, https://legumeinfo.org/): *M. truncatula* genotype A17 (JCVI Mt4.0v1), *G. max* (Wm82.a2.v1), *C. arietinum* (CDC Frontier, v1.0), *C. cajan* (v1.0), *P. vulgaris* (v1.0), and *L. japonicus* (v3). As the *Brassicaceae* lineage of *A. thaliana* was subjected to two additional and successive WGDs during lineage diversification [67], we also included the *Vitis vinifera* genome (v1.0) that did not undergo such additional WGDs [67]. All protein sequences are listed in Additional files 19, 20, 21. Proteins identified by BlastP search were then classified into the different TCS protein families depending on their domain composition. Protein domain composition of each protein was determined by a Hidden Markov Model (HMM; HMMER 3.0 [90]; e-value cut-off of 1e^− 10^) search against the Pfam domain database (http://pfam.xfam.org/; [91]). The domain composition of each TCS protein family is given in Additional file 17. For each protein, the identification of residues involved in histidine-aspartate phosphotransfer (H and/or D) was obtained after protein sequence alignment with a reference Arabidopsis protein sequence for which the position of these amino acids was previously functionally documented (At2g01830.1_AHK4/CRE1 for HKs, At3g21510.1_AHP1 for HPTs, At3g16857.2_ARR1 for RRBs, At5g62920.1_ARR6 for RRAs; www.arabidopsis.org).

The chromosomal distribution of all genes identified in the *M. truncatula* genome was established using the Phenogram software (http://visualization.ritchielab.psu.edu/phenograms/plot). Tandem and block duplicated genes were identified using the WGMapping whole genome mapping tool of the PLAZA 3.0 online database (https://bioinformatics.psb.ugent.be/plaza/versions/plaza_v3_dicots/; [92]).

### Phylogenetic and promoter analyses

Sequences were analyzed using Seaview (ver. 4.4.0; [93]) driving Muscle, GBlocks and PhyML. Full-length protein sequence alignments were generated with Muscle [94] and optimized with Gblocks [95]. Phylogenetic relationships were analyzed with a maximum likelihood approach. The tree was built with PhyML [96] using the LG substitution model [97] and four substitution rate categories. Support for each node was gained by approximate likelihood ratio tests (aLRT SH-like [96]). Phylogenetic trees were rooted with an *Ostreococcus tauri* HPT sequence (ID: 34527; https://genome.jgi.doe.gov) for HPT proteins and *A. thaliana* ARR22 (At3g04280) for RRs [75].

Promoter sequences (2.5 kb upstream the start codon) from all *M. truncatula* RRA encoding genes were retrieved from the *M. truncatula* genotype A17 genome (JCVI Mt4.0v1). The AGATHY *cis-*element motif, predicted to be bound by *A. thaliana* RRBs by [76] was searched in these promoters using the PlantPan 2.0 software (http://plantpan2.itps.ncku.edu.tw/promoter.php; [98].

### Expression data

Transcriptomic data were retrieved, using the *M. truncatula* Genome Database v4.0 (MtGD; http://www.medicagogenome.org/) IDs, on the *M. truncatula* Gene Expression Atlas (MtGEA) Affymetrix microarray database for the different plant organs ([68]; https://mtgea.noble.org/v3/), and on the Symbimics expression database (https://iant.toulouse.inra.fr/symbimics/) for RNAseq datasets from [65] for nodule zones and from [57] for the response to Nod factors in the root epidermis. All these experiments have been performed in the same genotype (Jemalong A17). Heat maps were built using conditional formatting in Excel (Microsoft) with a color scale from red (strongest expression) to white (weakest expression).

Hierarchical clustering of gene expression datasets retrieved from [60, 69] was performed using the MeV software (http://mev.tm4.org/), and the tree was build using Euclidean distances and an average linkage clustering.

For real-time RT-PCR analyses, total RNAs were extracted from frozen roots or nodules (8 days post- *S. meliloti* inoculation, or dpi) using the RNeasy plant mini kit (Qiagen, http://www.qiagen.com/). The first-strand cDNA was synthesized from 1 μg of total RNAs using the Superscript II first strand synthesis kit (Invitrogen, http://www.thermofisher.com/). Primer design was performed using the OligoPerfect™ Designer software (https://www.thermofisher.com/fr/fr/home/life-science/oligonucleotides-primers-probes-genes/custom-dna-oligos/oligo-design-tools/oligoperfect.html). Primer combinations showing a minimum amplification efficiency of 90% were retained (Additional file 18), and real-time RT-PCR reactions were performed using the Light Cycler Fast Start DNA Master SYBR Green I kit on a Light Cycler 480 apparatus according to manufacturer’s instructions (Roche). Cycling conditions were as follows: 95 °C for 10 min, and then 40 cycles at 95 °C for 15 s, 60 °C for 15 s, and 72°Cfor 15 s. PCR amplification specificity was verified using a dissociation curve. *MtRBP1* and *MtACTIN11* were previously selected as reference genes using the Genorm software (https://genorm.cmgg.be/).

## Additional files

Additional file 1: List of putative cytokinin receptors in the genome of *Arabidopsis thaliana*, *Vitis vinifera* and all studied legumes. For each chromosomal locus, the TCS protein name, as well as a previously published name when available, the protein length, the *A. thaliana* most closely related protein, and the conserved domains are listed. ^a^ [12]; ^b^ [99]; ^c^ [94]; ^d^ [54]; ^e^ [55]. (XLS 37 kb)

Additional file 2: Histidine kinases in *Arabidopsis thaliana*, *Cajanus cajan*, *Cicer arietinum*, *Glycine max*, *Lotus japonicus*, *Medicago truncatula, Phaseolus vulgaris, Vitis vinifera.* Phylogenetic tree of HKs based on full-length protein sequences from the seven-studied genomes. Protein sequences were aligned with the Muscle algorithm and the phylogenic tree was built with the Seaview software package. Numbers indicate the probability for each branch. (PDF 44 kb)

Additional file 3: List of putative ethylene receptors in the genome of *Arabidopsis thaliana*, *Vitis vinifera* and all studied legumes. For each chromosomal locus, the TCS protein name, as well as a previously published name when available, the protein length, the *A. thaliana* most closely related protein, and the conserved domains are listed. ^a^ [12]; ^b^ [99]; ^c^ [94]. (XLS 42 kb)

Additional file 4: List of putative AHK1 proteins in the genome of *Arabidopsis thaliana*, *Vitis vinifera* and all studied legumes. For each chromosomal locus, the TCS protein name, as well as a previously published name when available, the protein length, the *A. thaliana* most closely related protein, and the conserved domains are listed. ^a^ [12]; ^b^ [100]. (XLS 35 kb)

Additional file 5: List of putative CKI1 proteins in the genome of *Arabidopsis thaliana*, *Vitis vinifera* and all studied legumes. For each chromosomal locus, the TCS protein name, as well as a previously published name when available, the protein length, the *A. thaliana* most closely related protein, and the conserved domains are listed. ^a^ [12]; ^b^ [99]; ^c^ [100]. (XLS 32 kb)

Additional file 6: List of putative CKI2 proteins in the genome of *Arabidopsis thaliana*, *Vitis vinifera* and all studied legumes. For each chromosomal locus, the TCS protein name, as well as a previously published name when available, the protein length, the *A. thaliana* most closely related protein, and the conserved domains are listed. ^a^ [12]; ^b^ [99]; ^c^ [100]. (XLS 33 kb)

Additional file 7: List of putative HPT proteins in the genome of *Arabidopsis thaliana*, *Vitis vinifera* and all studied legumes. For each chromosomal locus, the TCS protein name, as well as a previously published name when available, the protein length, the *A. thaliana* most closely related protein, and the conserved domains are listed. ^a^ [12]; ^b^ [51]; ^c^ [99]; ^d^ [100]. (XLS 45 kb)

Additional file 8: Histidine Phosphotransfer proteins in *Arabidopsis thaliana*, *Cajanus cajan*, *Cicer arietinum*, *Glycine max*, *Lotus japonicus*, *Medicago truncatula, Phaseolus vulgaris, Vitis vinifera.* Phylogenetic tree of HPTs based on full-length proteins from the seven-studied genomes. Protein sequences were aligned with the Muscle algorithm and the phylogenic tree was built with the Seaview software package. Numbers indicate the probability for each branch. The tree was rooted on the HPT Ostta_34527 from *Ostreococcus tauri* [75]. (PDF 42 kb)

Additional file 9: Amino-acid substitution type and rate of the predicted H or D phosphoacceptor residue in HPT or RRB proteins. A. For the 78 legume HPT proteins identified, residue substitutions were analyzed, using MtHPT3 as a reference, at the H phosphoacceptor site (H77) and at the other H residues. B. For the 138 RRB proteins identified, residue substitutions were analyzed, using MtRRB3 as a reference, at the D phosphoacceptor site (D64) and at all other D residues. In both cases, D/N and D/E substitutions were analyzed separately whereas all other possible residue substitutions (“others”) were grouped together. (PDF 345 kb)

Additional file 10: List of putative RRBs in the genome of *Arabidopsis thaliana*, *Vitis vinifera* and all studied legumes. For each chromosomal locus, the TCS protein name, as well as a previously published name when available, the protein length, the *A. thaliana* most closely related protein, and the conserved domains are listed. ^a^ [12]; ^b^ [51]; ^c^ [99]; ^d^ [100]. (XLS 53 kb)

Additional file 11: Phylogenetic tree of Response Regulators in *Arabidopsis thaliana*, *Cajanus cajan*, *Cicer arietinum*, *Glycine max*, *Lotus japonicus*, *Medicago truncatula, Phaseolus vulgaris, Vitis vinifera.* Phylogenetic tree of RRs based on full-length proteins from the seven-studied genomes*.* Protein sequences were aligned with the Muscle algorithm and the phylogenic tree was built with the Seaview software package. Numbers indicate the probability for each branch. The tree was rooted on the ARR22 from *A. thaliana* [75]. (PDF 60 kb)

Additional file 12: List of putative RRCs in the genome of *Arabidopsis thaliana*, *Vitis vinifera* and all studied legumes. For each chromosomal locus, the TCS protein name, as well as a previously published name when available, the protein length, the *A. thaliana* most closely related protein, and the conserved domains are listed. ^a^ [12]; ^b^ [100]. (XLS 32 kb)

Additional file 13: List of putative Clock-RRs in the genome of *Arabidopsis thaliana*, *Vitis vinifera* and all studied legumes. For each chromosomal locus, the TCS protein name, as well as a previously published name when available, the protein length, the *A. thaliana* most closely related protein, and the conserved domains are listed. ^a^ [12]; ^b^ [100]. (XLS 34 kb)

Additional file 14: List of putative RRAs in the genome of *Arabidopsis thaliana*, *Vitis vinifera* and all studied legumes. For each chromosomal locus, the TCS protein name, as well as a previously published name when available, the protein length, the *A. thaliana* most closely related protein, and the conserved domains are listed. ^a^ [12]; ^b^ [51]; ^c^ [99]; ^d^ [100]; ^e^ [56]. (XLS 38 kb)

Additional file 15: Identification of predicted cytokinin response *cis-*elements in the promoter of *M. truncatula RRA* genes. Promoter sequences (2.5 kb upstream the start codon) from all *M. truncatula* RRA encoding genes were retrieved from the *M. truncatula* genome, and the number of the predicted AGATHY *A. thaliana* RRB binding motif was retrieved using the PlantPan 2.0 software. H stands for A/C/T and Y for C/T. (PDF 36 kb)

Additional file 16: Hierarchical clustering of the expression in roots and nodules of *Medicago truncatula* genes related to the Two Component System (TCS) signaling. Selected *M. truncatula* genome-wide expression datasets were used, corresponding to the Symbimics RNAseq database for roots, nodules and nodule zones [69], and for the root epidermis after a Nod Factors (NF) treatment [60]. Log2 expression values (deseq), normalized as described in the previously cited articles, were used for all TCS signaling genes identified in the *M. truncatula* genome to construct with the MeV software a heat-map based on Euclidean distances and average linkage clustering. The color scale ranges from red (no expression) to blue (strongest expression). A color code was additionally used for gene names corresponding to the different TCS protein families: in black, CHKs (CHASE domain containing Histidine Kinases); in green, HPTs (Histidine PhosphoTranfert proteins); in blue, RRBs (Type-B Response Regulators); and in red, RRAs (Type-A Response Regulators). Non-canonical proteins are labelled with a blue dot, and the bracket indicates the core cytokinin signaling identified in the study. Nodule zones were defined as in [69]: ZI, meristematic zone; ZIId and ZIIp, distal and proximal differentiation/rhizobial infection zones; IZ, inter-zone II/III; ZIII, nitrogen fixation zone. NF, Nod Factor treatment as described in [60]. (XLSX 9 kb)

Additional file 17: Domain composition of the different TCS protein families. For each TCS protein family, the domain composition is given. The Pfam ID is indicated for each domain. (XLS 28 kb)

Additional file 18: List of primers used. (XLSX 14 kb)

Additional file 19: Sequences of all Histidine Kinases (HKs) proteins. Sequences are listed in the fasta format. (TXT 122 kb)

Additional file 20: Sequences of all Histidine PhosphoTranfer (HPT) proteins. Sequences are listed in the fasta format. (TXT 14 kb)

Additional file 21: Sequences of all Response Regulators (RR) proteins. Sequences are listed in the fasta format. (TXT 140 kb)
