# Supplementary figures and images for: Diversification of cytokinin phosphotransfer signaling genes in Medicago truncatula and other legume genomes
Source: BMC Genomics. 2019 May 14;20:373. doi: 10.1186/s12864-019-5724-z (PMC6518804; doi:10.1186/s12864-019-5724-z)

Cytokinin receptors

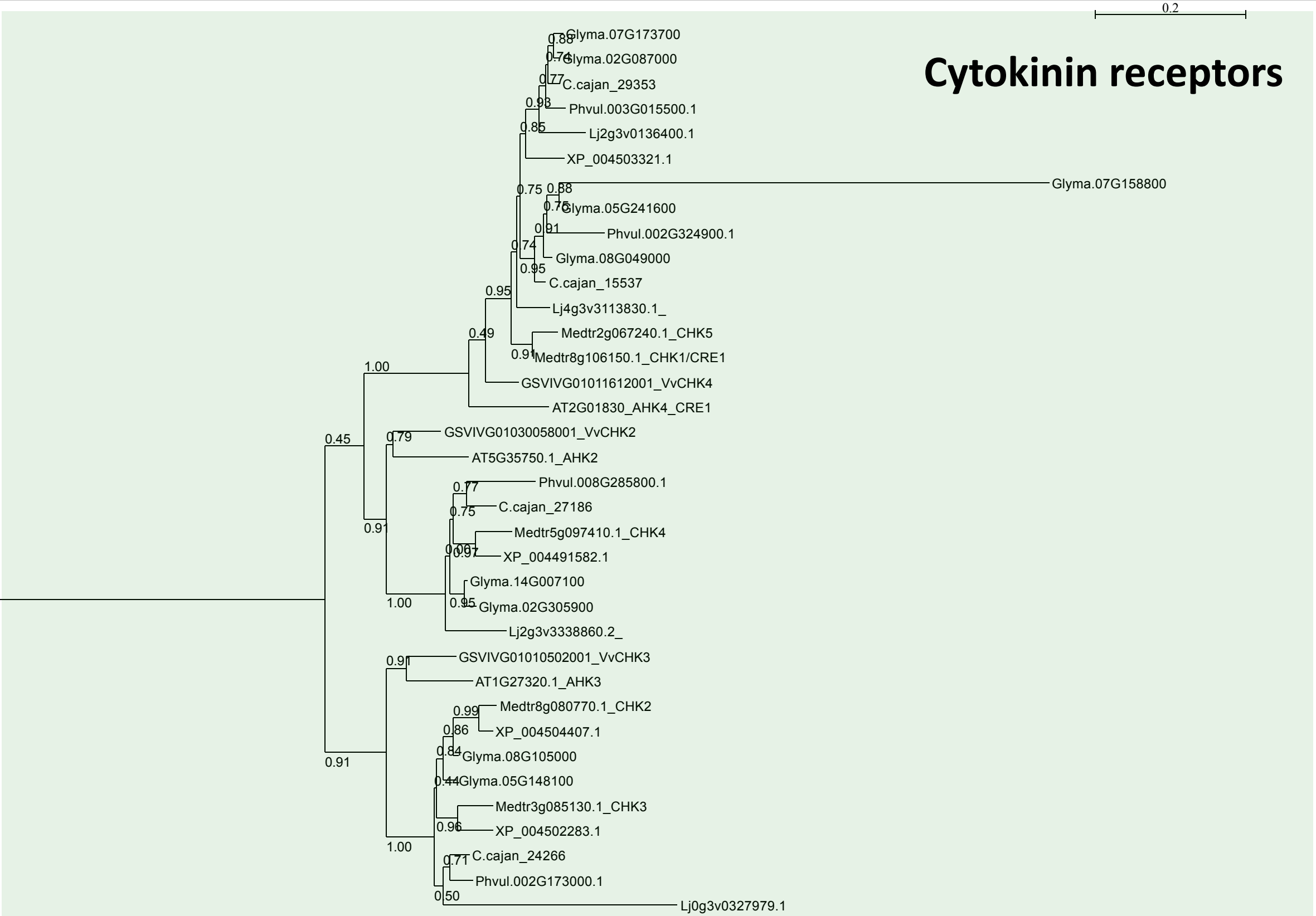

Osmosensors

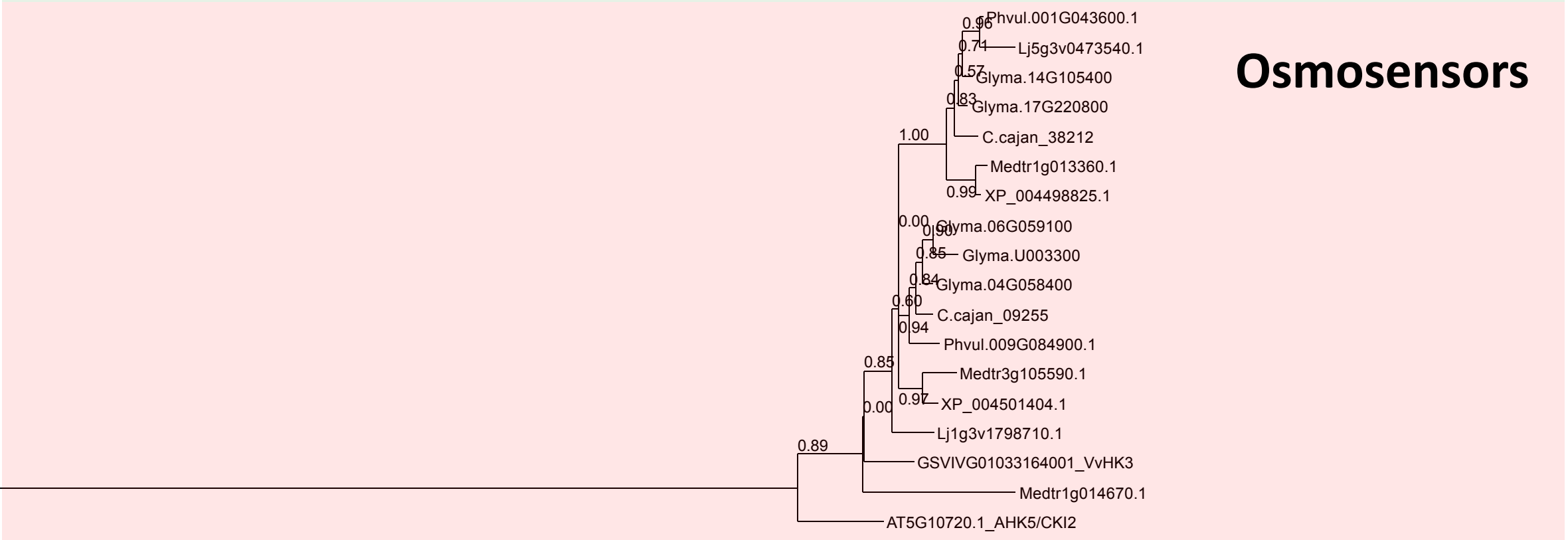

Ethylene receptors

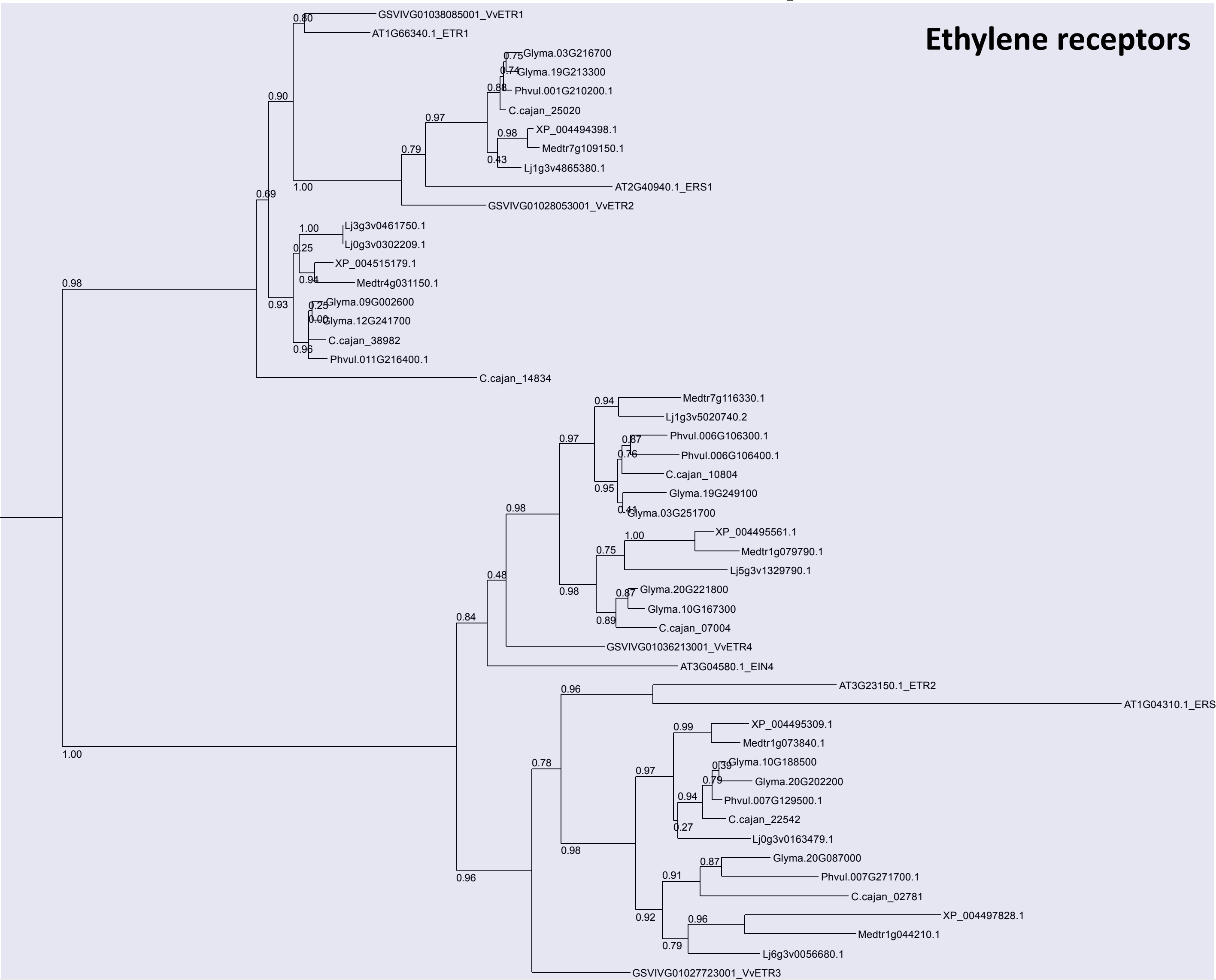

Supplement: Supplementary file 2 — Histidine kinases in Arabidopsis thaliana, Cajanus cajan, Cicer arietinum, Glycine max, Lotus japonicus, Medicago truncatula, Phaseolus vulgaris, Vitis vinifera. Phylogenetic tree of HKs based on full-length protein sequences from the seven-studied genomes. Protein sequences were aligned with the Muscle algorithm and the phylogenic tree was built with the Seaview software package. Numbers indicate the probability for each branch. (PDF 44 kb) [file 12864_2019_5724_MOESM2_ESM.pdf]

HPT-N

HPT-H

HPT-X

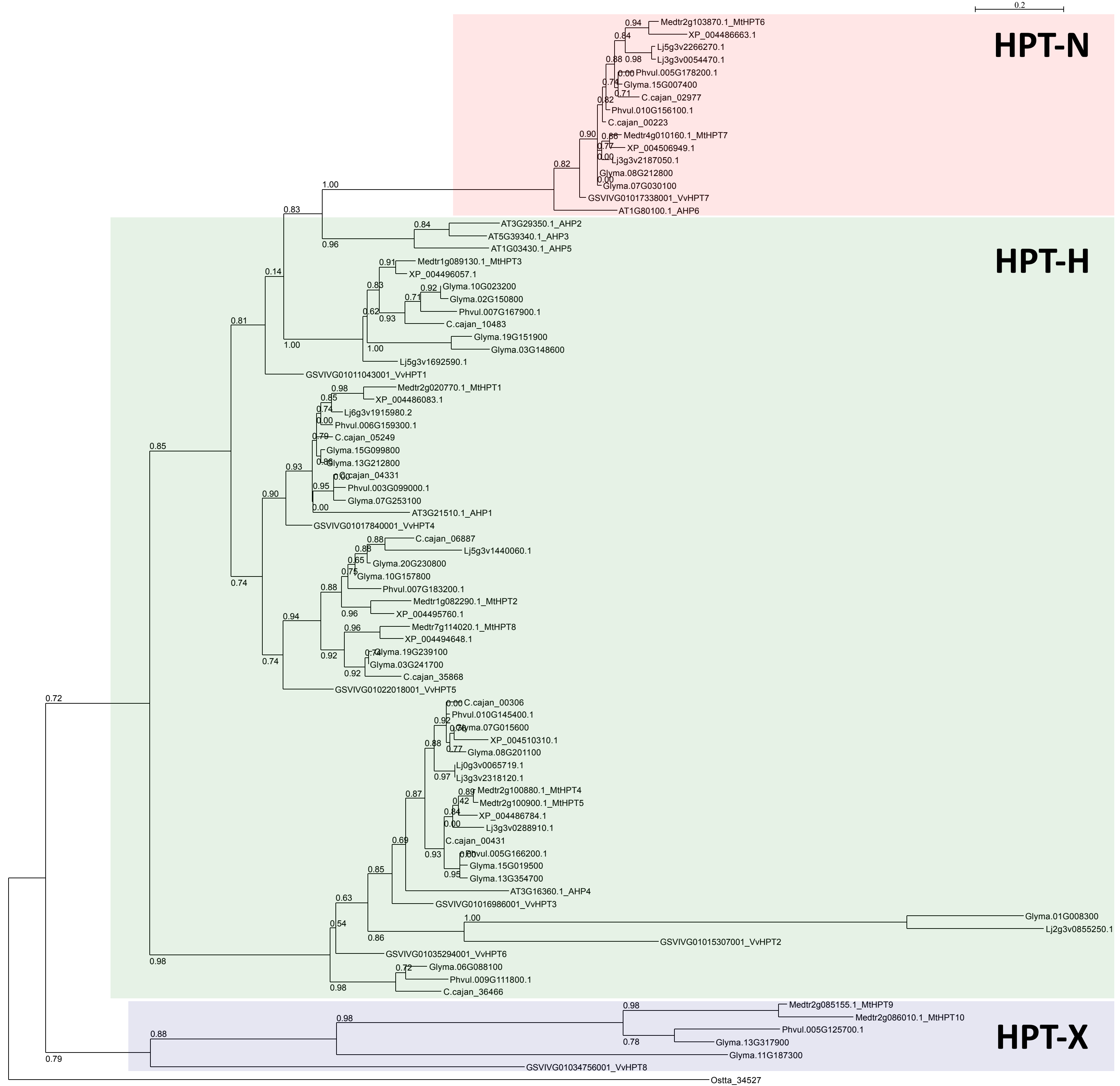

Supplement: Supplementary file 8 — Histidine Phosphotransfer proteins in Arabidopsis thaliana, Cajanus cajan, Cicer arietinum, Glycine max, Lotus japonicus, Medicago truncatula, Phaseolus vulgaris, Vitis vinifera. Phylogenetic tree of HPTs based on full-length proteins from the seven-studied genomes. Protein sequences were aligned with the Muscle algorithm and the phylogenic tree was built with the Seaview software package. Numbers indicate the probability for each branch. The tree was rooted on the HPT Ostta_34527 from Ostreococcus tauri [75]. (PDF 42 kb) [file 12864_2019_5724_MOESM8_ESM.pdf]

A

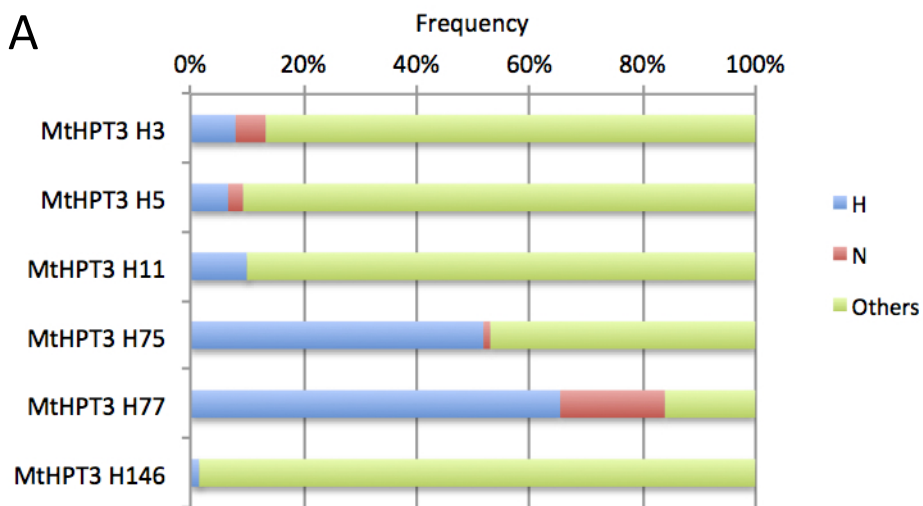

B

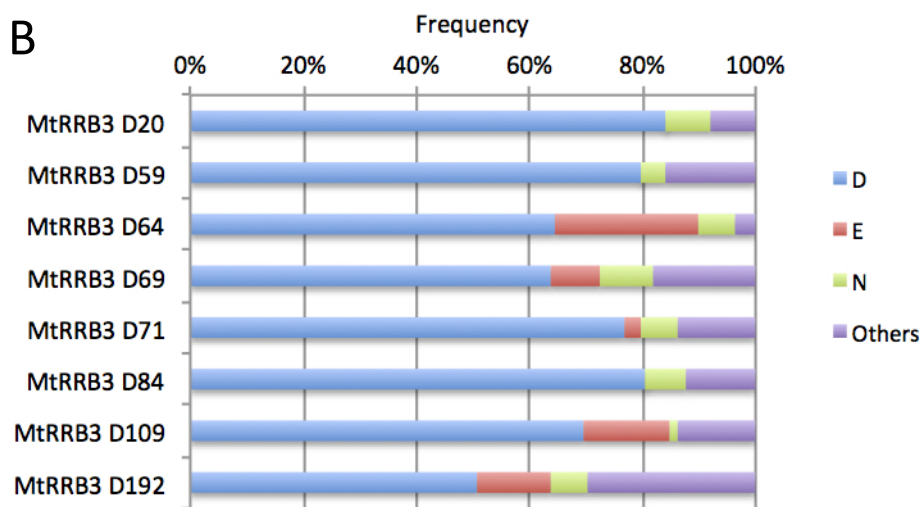

Supplement: Supplementary file 9 — Amino-acid substitution type and rate of the predicted H or D phosphoacceptor residue in HPT or RRB proteins. A. For the 78 legume HPT proteins identified, residue substitutions were analyzed, using MtHPT3 as a reference, at the H phosphoacceptor site (H77) and at the other H residues. B. For the 138 RRB proteins identified, residue substitutions were analyzed, using MtRRB3 as a reference, at the D phosphoacceptor site (D64) and at all other D residues. In both cases, D/N and D/E substitutions were analyzed separately whereas all other possible residue substitutions (“others”) were grouped together. (PDF 345 kb) [file 12864_2019_5724_MOESM9_ESM.pdf]

## Clock-RRs

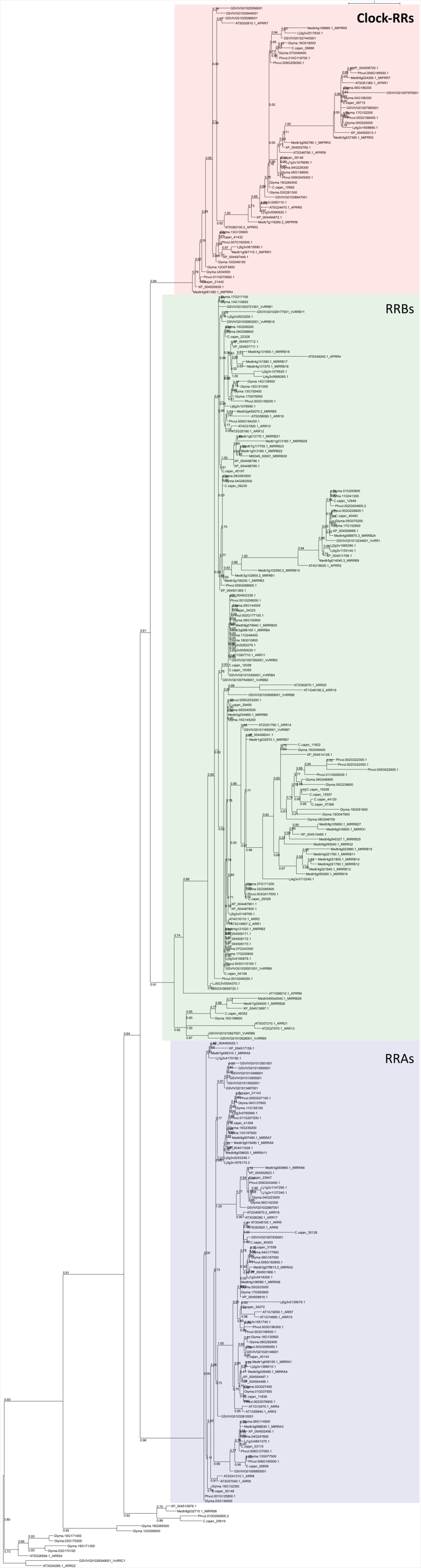

Supplement: Supplementary file 11 — Phylogenetic tree of Response Regulators in Arabidopsis thaliana, Cajanus cajan, Cicer arietinum, Glycine max, Lotus japonicus, Medicago truncatula, Phaseolus vulgaris, Vitis vinifera. Phylogenetic tree of RRs based on full-length proteins from the seven-studied genomes. Protein sequences were aligned with the Muscle algorithm and the phylogenic tree was built with the Seaview software package. Numbers indicate the probability for each branch. The tree was rooted on the ARR22 from A. thaliana [75]. (PDF 60 kb) [file 12864_2019_5724_MOESM11_ESM.pdf]

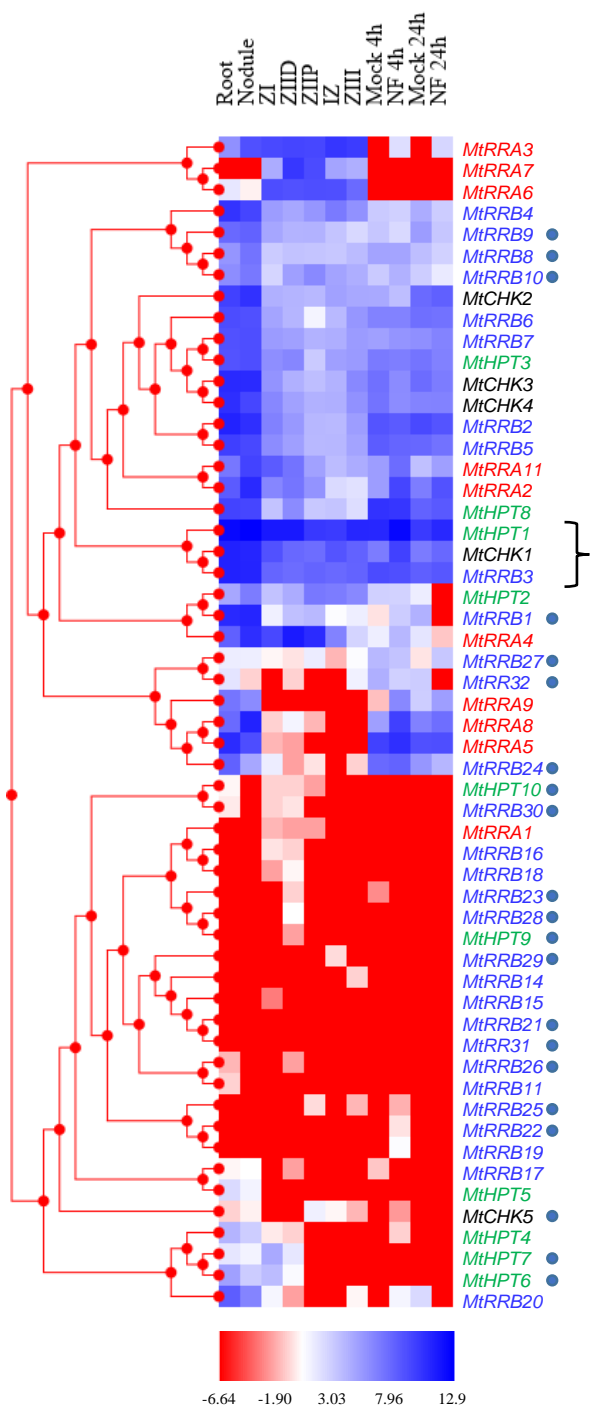

Supplement: Supplementary file 15 — Identification of predicted cytokinin response cis-elements in the promoter of M. truncatula RRA genes. Promoter sequences (2.5 kb upstream the start codon) from all M. truncatula RRA encoding genes were retrieved from the M. truncatula genome, and the number of the predicted AGATHY A. thaliana RRB binding motif was retrieved using the PlantPan 2.0 software. H stands for A/C/T and Y for C/T. (PDF 36 kb) [file 12864_2019_5724_MOESM15_ESM.pdf]
